# Divergence‐free tangential finite element methods for incompressible flows on surfaces

**DOI:** 10.1002/nme.6317

**Published:** 2020-02-18

**Authors:** Philip L. Lederer, Christoph Lehrenfeld, Joachim Schöberl

**Affiliations:** ^1^ Institute for Analysis and Scientific Computing TU Wien Vienna Austria; ^2^ Institute for Numerical and Applied Mathematics University of Göttingen Göttingen Germany

**Keywords:** divergence‐conforming finite elements, incompressible Navier‐Stokes equations, Piola transformation, surface PDEs, tangential vector field

## Abstract

In this work we consider the numerical solution of incompressible flows on two‐dimensional manifolds. Whereas the compatibility demands of the velocity and the pressure spaces are known from the flat case one further has to deal with the approximation of a velocity field that lies only in the tangential space of the given geometry. Abandoning *H* ^1^‐conformity allows us to construct finite elements which are—due to an application of the Piola transformation—exactly tangential. To reintroduce continuity (in a weak sense) we make use of (hybrid) discontinuous Galerkin techniques. To further improve this approach, $H({\text{div}}_{\Gamma })$‐conforming finite elements can be used to obtain exactly divergence‐free velocity solutions. We present several new finite element discretizations. On a number of numerical examples we examine and compare their qualitative properties and accuracy.

## 1 INTRODUCTION

Partial differential equations (PDEs) that are posed on curved surfaces play an important role in several applications in engineering, physics, and mathematics. Surface PDEs describing flows on surfaces appear for instance in the modeling of emulsions, foams, and biological membranes, cf Slattery et al^1^ or Brenner,^2^ or liquid crystals, cf de Gennes and Prost^3^ or Napoli and Vergori.^4^ The numerical treatment of these PDEs gained an increasing amount of attention in the field of numerical simulations and numerical analysis in the last two decades. In this work we consider vector‐valued PDEs for viscous incompressible flows on surfaces that are immersed in the three‐dimensional space.

A main source of difficulty for vector‐valued PDEs on surfaces is the fact that the unknown vector field is typically *tangential*, that is, for a two‐dimensional manifold Γ embedded in $R^{3}$ the unknown field is only two‐dimensional. In recent works almost exclusively [*H* ^1^(Γ)]^3^‐conforming finite elements have been used to approximate the unknown tangential vector field. Tangential vector fields are only weakly imposed through the variational formulation. In this work we follow a different approach: we abandon continuity of the finite elements. This loss of conformity, however, allows us to construct exactly tangential vector fields. This is achieved by mapping finite element functions from the two‐dimensional reference element by a straight‐forward generalization of the well‐known *Piola transformation*. This guarantees that the resulting (possibly higher order) vectorial basis functions are *exactly tangential* to the surface. This specifically means that no additional enforcement of the tangentiality condition is needed to be enforced through the variational formulation. One could say that we trade one structure property (continuity) for the other (tangential vector fields). To deal with the missing continuity we apply well‐established techniques from the flat case: discontinuous Galerkin (DG) methods and variants such as the hybrid DG (HDG) methods.

It turns out that we do not have to abandon continuity completely, but can preserve continuity at least for the conormal component, that is, the in‐plane normal component across element interfaces, resulting in $H({\text{div}}_{\Gamma })$‐conforming finite elements. These finite elements in conjunction with suitable (hybrid) DG techniques have been proven to be excellent discretizations for incompressible fluid flows in the flat case due to beneficial properties such as exactly divergence‐free solutions, pressure robustness, and energy stability. These properties can easily be transferred to the case of surface Stokes and surface Navier‐Stokes equations as we will explain in the sequel of this article.

### 1.1 State of the art

Let us briefly give an overview on the state of the art in the literature. Initially surface finite element methods for *scalar* PDEs have been introduced in the seminal work by Dziuk,^5^ we also refer to the survey articles by Dziuk and Elliott^6^ and Bonito et al.^7^ The use and analysis of *H* ^1^(Γ)‐conforming surface finite elements has been extended to higher order discretizations and adaptive schemes by Demlow et al,^8^, ^9^ including the analysis of geometry errors.^10^ In the last decade the extension to non‐(*H* ^1^(Γ)‐)conforming surface finite elements has been done in several works, cf for instance Dedner et al^11^, ^12^ and eventually also HDG formulations have been considered by Cockburn and Demlow.^13^ In all these works an explicit surface mesh is used. A different approach is based on a mesh of the surrounding 3D space and a level set description of the surface. In the “TraceFEM” the trace on the level set surface of finite element functions of this background mesh are used for the approximation of the solution. The method was introduced by Olshanskii et al.^14^ Sometimes the method is also known as “CutFEM”, cf, for example, Burman et al.^15^ We also refer to the overview article on TraceFEM by Olshanskii and Reusken.^16^

Mixed formulations of the surface Poisson problem can be seen as an intermediate step towards vector‐valued PDEs as they involve the vector‐valued surface flux that is approximated separately from the primal unknown, resulting in a system of first order surface PDEs. These mixed formulations have been considered for instance by Rognes et al^17^ and—as part of their mixed formulations of DG and HDG methods—by Antonietti et al^12^ and Cockburn and Demlow.^13^ Here, tangential surface finite elements are constructed for the flux. Primal DG formulations in the context of TraceFEM/CutFEM have also been considered by Burman et al.^18^ In the works by Rognes et al^17^ and Cockburn et al^13^ the construction of the spaces is based on the Piola transformation, resulting in (broken) surface Raviart‐Thomas spaces as considered already by Nedelec^19^ and Bendali.^20^ Let us note that the analysis of mixed Poisson formulations including variational crimes in a general framework has been considered in Holst and Stern.^10^ In all these works, where the primal unknown is scalar, an isoparametric geometry approximation, that is, using order *k* for approximating the scalar unknown and order *k* for the geometry approximation is sufficient to obtain order *k* + 1 error estimates in the *L* ^2^‐norm. Only for the superconvergence property in the HDG method by Cockburn and Demlow^13^ an increased geometry order is necessary.

While scalar problems on surfaces and their numerical treatment seem to be well understood vector‐valued problems have drawn an increased interest recently. Relevant models of viscous fluidic surfaces based on 3D Cartesian differential operators are described in Jankuhn et al^21^ and Koba et al.^22^ In the context of FME these PDEs require vector‐valued finite element spaces on surfaces that are tangential. Starting with the Vector Laplacian surface finite elements that are [*H* ^1^(Γ)]^3^‐conforming, that is, three‐dimensional, and impose the tangential condition through the variational formulation have been proposed by Hansbo et al.^23^ In that article, a penalty formulation and a Lagrange multiplier‐based formulation are considered to drive the normal component of the discrete approximation to zero. Further, it is already observed that—in contrast to the scalar problem—an isoparametric discretization is not sufficient to preserve optimal order *L* ^2^‐errors. In Jankuhn et al,^24^, ^25^ similar approaches are considered and analyzed in the context of TraceFEM discretizations. Hansbo et al^26^ extended their approach to Darcy problems on surfaces using [*H* ^1^(Γ)]^3^‐conforming (low‐order) surface FEM. The surface Stokes problem based on a velocity‐pressure formulation has been discretized using TraceFEM based on a stabilized P1‐P1 discretization in Olshanskii et al.^27^ In the very recent article by Olshanskii et al^28^ the TraceFEM with a P2‐P1 discretization has been considered. Also only very recently Bonito et al^29^ presented a low‐order $H({\text{div}}_{\Gamma })$‐conforming discretization for the surface Stokes problem on *C* ^4^ smooth closed surfaces with a focus on the numerical treatment of *killing fields*. Their approach to discretize the surface Stokes problem is similar to the discretizations that we treat for the surface Navier‐Stokes equations in this article. Similar methods to Hansbo et al^23^ have been proposed in Nestler et al,^30^, ^31^ where vector‐ and tensor‐valued surface PDE models are considered.

A vorticity formulation has been considered for the surface Stokes problem in Reuther et al and Reusken^32^, ^33^, ^34^ where the explicit construction of tangential vector fields is circumvented. Vorticity formulations have also been used to solve the surface Navier‐Stokes equations in Azencot et al^35^ and Nitschke et al.^36^ Velocity‐pressure formulations for the surface Navier‐Stokes problem have been recently considered using higher order [*H* ^1^(Γ)]^3^‐conforming surface FEM by Reuther and Voigt^37^ (low order) and by Fries^38^ (higher order) and based on a low‐order TraceFEM discretization with penalty by Olshanskii and Yushutin.^39^ We also mention the numerical approaches based on discrete exterior calculus in Nitschke et al^33^ and spectral methods in Gross and Atzberger.^40^

In all the previous works either closed smooth surfaces or at least smooth surfaces with boundaries, for example, in Fries,^38^ have been considered. The case of only piecewise smooth geometries has not been addressed in the finite element literature so far to the best of our knowledge.

As we will base our discretization for the surface Navier‐Stokes equations on $H({\text{div}}_{\Gamma })$‐conforming elements, let us also briefly mention previous works on $H({\text{div}}_{\Gamma })$‐conforming methods in the plane. In the context of DG discretizations Cockburn et al^41^ were the first to realize that energy stability and local mass conservation for DG methods is only achieved for $H({\text{div}}_{\Gamma })$‐conforming finite elements which result in pointwise divergence‐free solutions. We extended this idea to HDG methods and considered several extensions and improvements and evaluated the computational efficiency of the resulting methods in the works^42^ to,^48^ cf also the discussion in Section 3.5 below.

### 1.2 Content and structure

In this article we introduce non‐(*H* ^1^(Γ)‐)conforming finite elements for incompressible surface flows, starting from the Vector Laplacian to the unsteady surface Navier‐Stokes equations. We present different DG and HDG discretizations—most of which are new—and compare them to [*H* ^1^(Γ)]^3^‐conforming methods. The use of $H({\text{div}}_{\Gamma })$‐conforming FME results in pointwise divergence‐free and exactly tangential solutions. Our methods are high order accurate but allow for surfaces which are only piecewise smooth. Moreover, arbitrary surface meshes can be dealt with, that is, an exact geometry description does not need to be known.

Model problems are presented in Section 2 before several numerical schemes with tangential finite elements are introduced in Section 3. Several numerical examples for the different problems and discretizations are presented and discussed in Section 4 before we conclude the manuscript.

## 2 MODEL PROBLEMS ON THE SURFACE

### 2.1 Notation and surface differential operators

Let Γ be a sufficiently smooth connected two‐dimensional stationary and oriented surface embedded in $R^{3}$. At every point *x*∈Γ we denote by ***n*** (*x*) a uniquely oriented unit normal vector, and by *P*(*x*)=*I*−***n*** (*x*) ***n*** (*x*)^*T*^, with the identity matrix *I*, the corresponding orthogonal projection onto the tangential plane of Γ at *x*. In this work we assume that there exists a sufficiently smooth extension into the neighborhood 𝒪(Γ) of Γ which induces a projection p:𝒪(Γ)→Γ. Assume a given scalar‐valued, sufficiently smooth function ϕ:Γ→R, and let $\phi^{p}=\phi \circ p:𝒪(\Gamma )\to R$ denote its extensions to the neighborhood 𝒪(Γ) of Γ. Then, we define the *scalar surface gradient* through the gradient of *ϕ* ^*p*^ in the embedding space and the projection onto the tangential plane. Hence, for any *x*∈ Γ we have

$$\begin{array}{ll} \nabla_{\Gamma }\phi (x):=P(x)\nabla \phi^{p}(x), & \; \end{array}$$

where ∇*ϕ* ^*p*^ is the column vector consisting of all partial derivatives. As a direct consequence we realize that the scalar surface gradient lies in the tangential plane of Γ. Further, let us note that the surface gradient is independent of the concrete choice of the projection *p*. In the same manner let $u={\left(u_{1},u_{2},u_{3}\right)}^{T}:\Gamma \to R^{3}$ be a given vector‐valued and sufficiently smooth function and denote by $u^{p}:𝒪(\Gamma )\to R^{3}$ its extension to the neighborhood. According to the above definition we can define the componentwise surface gradient through ∇*u* ^*p*^ *P*, where $\nabla u^{p}={\left(\nabla u_{1}^{p},\nabla u_{2}^{p},\nabla u_{3}^{p}\right)}^{T}$ is the standard Jacobian matrix of *u* ^*p*^. Hence, ∇*u* ^*p*^ *P* is the matrix where each row gives the scalar surface gradient of the components of *u* ^*p*^. We define another operator called the *tangential surface gradient* by applying an additional projection from the left

$$\begin{array}{ll} \nabla_{\Gamma }u(x):=P(x)\nabla u^{p}(x)P(x)\;\forall x\in \Gamma . & \; \end{array}$$

Note, that in the literature this operator is usually known as the covariant derivative on Γ. We are now able to state the symmetric surface strain tensor which is—following Gurtin and Murdoch^49^—defined as

(1) $$\varepsilon_{\Gamma }(u):=\frac{1}{2}(\nabla_{\Gamma }u+\nabla_{\Gamma }u^{T}),$$

and the surface divergence operator

$$\begin{array}{ll} {\text{div}}_{\Gamma }u & :=\mathrm{tr}(\nabla_{\Gamma }u).\; \end{array}$$

As usual the divergence operator ${\text{div}}_{\Gamma }$ applied on a matrix valued function σ reads as the rowwise divergence.

So far we assumed that *ϕ* and *u* are sufficiently smooth so that the above differential operators exist in a pointwise sense on Γ. We can generalize to the notion of weak derivatives in the usual sense. For instance we define the weak gradient *g* ^*ϕ*^∈[*L* ^2^(Γ)]^3^ (if it exists) of a given function *ϕ*∈*L* ^2^(Γ), as the function that fulfills $\int_{\Gamma }g^{\phi }\cdot v=-\int_{\Gamma }\phi \;{\text{div}}_{\Gamma }\;v$ for all $v\in {\left[C_{0}^{\infty }(\Gamma )\right]}^{3}$.

In the next three subsections we introduce the surface PDE problems considered in this work. To this end let *f*∈[*L* ^2^(Γ)]^3^ such that *f*· ***n*** =0 on Γ be a given force vector.

### 2.2 Vector‐valued elliptic problem on Γ:

We seek for a solution $u:\Gamma \to R^{3}$ with *u*· ***n*** =0 on Γ of the second order PDE given by

(2a) $$\begin{array}{ll} -P\;{\text{div}}_{\Gamma }(\varepsilon_{\Gamma }(u))+u & =f\;\text{in}\;\Gamma , \end{array}$$

(2b) $$\begin{array}{ll} \;u & =0\;\text{on}\;\partial \Gamma .\; \end{array}$$

For the ease of presentation we only consider homogeneous Dirichlet boundary conditions in this part but the introduced methods can all be extended to more general boundary conditions, as demonstrated in the numerical examples. Further note that in the case of a closed surface (∂Γ=∅) no boundary conditions are prescribed. Let *V*:={*u*∈[*H* ^1^(Γ)]^3^:*u*=0 on ∂Γ} with the norm $\|u{\|}_{1}^{2}:=\|\nabla_{\Gamma }u{\|}_{L^{2}(\Gamma )}^{2}+\|u{\|}_{L^{2}(\Gamma )}^{2}$, and let

$$\begin{array}{ll} V_{\tau }(\Gamma ):=\{u\in V:u\cdot n=0\;\text{on}\;\Gamma \}. & \; \end{array}$$

Following Groß et al,^24^ the variational formulation of (2) is given by: Find *u*∈*V*_**τ**_ such that

(3) $$\begin{array}{ll} a(u,v)+m(u,v)=f(v)\;\forall v\in V_{\tau }, & \; \end{array}$$

where

$$\begin{array}{ll} a(u,v):=\int_{\Gamma }\varepsilon_{\Gamma }(u):\varepsilon_{\Gamma }(v)dx,\;m(u,v):=\int_{\Gamma }u\cdot vdx,\;f(v):=\int_{\Gamma }f\cdot vdx. & \; \end{array}$$

While finite element discretization of such variational problems are well understood on a flat surface, the tangential constraint *u*· ***n*** =0 on Γ makes the construction of an appropriate numerical scheme on surfaces much more difficult. In Section 3 we discuss a natural approach how to deal with this challenge.

Remark 1 Note that the above variational formulation can also be defined on piecewise smooth connected surfaces. Then the strong form of the PDE is given by Equation (2) defined on each (smooth) sub domain and continuity conditions of the trace and the normal fluxes at the common interfaces. In Section 4.1.3 we demonstrate that our methods are applicable for such problems.

### 2.3 Stationary Stokes equations on Γ

We consider a Newtonian fluid on Γ, see Jankuhn et al^21^ and Koba et al,^22^ and assume for the ease of representation that ∂Γ≠∅, see Remark 2. Adding the incompressibility constraint and the pressure as Lagrange multiplier we now seek for a solution $u:\Gamma \to R^{3}$ with *u*· ***n*** =0 on Γ and p:Γ→R such that

(4a) $$\begin{array}{ll} -2\nu P\;{\text{div}}_{\Gamma }(\varepsilon_{\Gamma }(u))+\nabla_{\Gamma }p & =f\;\text{in}\;\Gamma , \end{array}$$

(4b) $$\begin{array}{ll} \;{\text{div}}_{\Gamma }(u) & =0\;\text{in}\;\Gamma , \end{array}$$

(4c) $$\begin{array}{ll} \;u & =0\;\text{on}\;\partial \Gamma .\; \end{array}$$

Here, (4a) can also be read as $-P\;{\text{div}}_{\Gamma }\;\sigma (u)=f$ with the stress tensor σ(*u*,*p*)=−*pP*+2*νε*_Γ_(*u*) where ν is the kinematic viscosity. Following the derivation in Fries^38^ the weak formulation is given by

$$\begin{array}{lll} 2\nu a(u,v)+b(v,p) & =f(v) & \;\forall v\in V_{\tau },\\ b(u,q) & =0 & \forall q\in Q, \end{array}$$

with $b(u,q)=\int_{\Gamma }{\text{div}}_{\Gamma }(u)qdx$, and the pressure space $Q:=\{q\in L^{2}(\Gamma ):\int_{\Gamma }q=0\}$. Beside the crucial constraint that *u* is in the tangential space of Γ we now further have to deal with the divergence constraint. In particular this plays a key role in the discretization as one has to find compatible function spaces for the discrete velocity and the pressure spaces.

Remark 2 As the variational formulation of the standard stationary (flat) Stokes equations is defined without a mass bilinear form *m*(*u*,*v*) it demands further constraints to filter out the possibly nontrivial kernel of the composite bilinear form involving *a*(·,·) and *b*(·,·). On surfaces with a boundary sufficient constraints can be obtained from suitable boundary conditions. However, for the surface Stokes equations on closed surfaces the nontrivial kernel, the so‐called killing fields, need to be taken care of to guarantee uniqueness. For a more detailed discussion we refer to Bonito et al^29^ and Reuther and Voigt.^37^

### 2.4 Unsteady Navier‐Stokes equations on Γ

We extend the Stokes model to the fully unsteady Navier‐Stokes case, that is, we seek for a solution $u:\Gamma \times (0,T]\to R^{3}$ with *u*· ***n*** =0 on Γ and p:Γ×(0,T]→R such that

(5a) $$\begin{array}{ll} \partial_{t}u-2\nu P{\;\text{div}}_{\Gamma }(\varepsilon_{\Gamma }(u))+(u\cdot \nabla_{\Gamma })u+\nabla_{\Gamma }p & =f\;\text{on}\;\Gamma ,t\in (0,T], \end{array}$$

(5b) $$\begin{array}{ll} \;{\text{div}}_{\Gamma }(u) & =0\;\text{on}\;\Gamma ,t\in (0,T], \end{array}$$

(5c) $$\begin{array}{ll} \;ℬ(u) & =g\;\text{on}\;\partial \Gamma ,t\in (0,T], \end{array}$$

(5d) $$\begin{array}{ll} \;u & =u_{0}\;\text{on}\;\Gamma \times \{0\}, \end{array}$$

with *T*>0, a given boundary differential operator ℬ, boundary values *g*, and initial values *u*_0_. Beside the difficulties already discussed in the Stokes setting, the challenging part now is to deal with the nonlinear convection and the time derivative.

## 3 CONSTRUCTION OF TANGENTIAL FME

In this section we focus on the derivation of new tangential DG schemes for the problems introduced in Section 2. After introducing some basic notation in Section 3.1, we concentrate on rather standard DG versions first in Sections 3.2–3.4. Benefits of choosing a $H({\text{div}}_{\Gamma })$‐conforming formulations are discussed in Section 3.5. In Section 3.6 we introduce similar HDG formulations and explain their computational advantage over the standard DG formulations.

### 3.1 Basic ingredients and motivation

In this section we discuss a natural approach for the construction of tangential vector fields. To this end we summarize the findings of the works.^17^, ^50^ Let ${𝒯}_{h}$ be a consistent triangulation of the smooth manifold Γ into curved triangles such that for every element $T\in {𝒯}_{h}$ there exists a not degenerated polynomial mapping Φ_*T*_ of order *k*_*g*_ from the reference element

$$\begin{array}{ll} \hat{T}:=\{(x,y)\in R^{2}:0\leq x+y\leq 1\}. & \; \end{array}$$

to the physical element *T*, that is, $\Phi_{T}:\hat{T}\to T$. With respect to this triangulation, we write $\Gamma_{h}:=\cup_{T\in {𝒯}_{h}}T$ for the corresponding locally smooth discrete manifold. In the following we will use the notation *ε*_Γ_, *P* and ∇_Γ_ also for operations w.r.t. to Γ_*h*_ (instead of Γ). Further, we define the set ${ℱ}_{h}$ as the union of all element interfaces. We assume that ${𝒯}_{h}$ is shape regular and quasiuniform, thus there exists a mesh size *h* with h≃diam(T) for all $T\in {𝒯}_{h}$. We denote by $F_{T}=\Phi_{T}^{\prime }\in R^{3\times 2}$ the Jacobian of the finite element mapping and remind the reader that the columns of *F*_*T*_ span the tangential space for each point in *T*. In the following, we will drop the subscript *T* in the transformation Φ_*T*_ and the derived quantities such as the Jacobian *F*_*T*_ unless the association to the element *T* shall be highlighted. Next, we write

$$\begin{array}{ll} F^{-1}={\left(F^{T}F\right)}^{-1}F^{T} & \; \end{array}$$

for the Moore‐Penrose pseudo inverse of the Jacobian *F*, and set $J:=\sqrt{\det(F^{T}F)}$ as the functional determinant. Now let $\hat{\phi }$ be a differentiable function defined on the reference element $\hat{T}$ and let $x=\Phi (\hat{x})\in T$ for all points $\hat{x}\in \hat{T}$. Using the classical pull back we define a function *ϕ* on *T* by

$$\begin{array}{ll} \phi (x)=\hat{\phi }(\hat{x}). & \; \end{array}$$

Following the ideas of Rognes et al,^17^ this pullback allows us to calculate the surface gradient of the function *ϕ* by

$$\begin{array}{ll} \nabla_{\Gamma }\phi (x)=F^{-T}\nabla \hat{\phi }(\hat{x}). & \; \end{array}$$

As *F* ^−*T*^=*F*(*F* ^*T*^ *F*)^−*T*^, the above equation shows that the gradient ∇*ϕ* is a linear combination of the two tangent vectors **τ**_1_=*Fe*_1_, **τ**_2_=*Fe*_2_ and hence lies in the tangent space as expected from differential geometry, see for example Dziuk and Elliott.^6^ The above construction allows us to define an *H* ^1^‐conforming finite element space of order *k* on the surface Γ_*h*_ by

$$\begin{array}{ll} S_{h}^{k}:=\{w\in C^{0}(\Gamma_{h}):\forall T\in {𝒯}_{h}\;\exists ŵ\in P^{k}(\hat{T})\;\text{s.t.}\;w{|}_{T}\circ \Phi_{T}=ŵ\}. & \; \end{array}$$

where $P^{k}(\hat{T})$ is the space of polynomials up to degree *k* on $\hat{T}$. Whereas the above technique allows an easy construction of a scalar approximation space, the problems (2), (4), and (5) demand for vector‐valued approximation spaces. In particular we aim for a space that can handle the constraint *u*· ***n*** =0 in a proper way. Obviously, the simple product space $S_{h}^{k}\times S_{h}^{k}\times S_{h}^{k}$ is not convenient and we need a different construction. The solution to this is given by using the Piola transformation instead of the classical pull back. Originally, thus on flat surfaces, this mapping is used for the construction of H(div)‐conforming finite element spaces as it preserves the normal components on element interfaces. To this end let û be a vector‐valued function on $\hat{T}$, then we define on *T* the function

(6) $$\begin{array}{ll} u(x)={𝒫}_{T}(û)(x):=\frac{1}{J}Fû(\hat{x}). & \; \end{array}$$

Due to the multiplication with *F*, we again see that the constructed vector field *u* lies in the tangential plane of *T*. This finding is the key argument and motivation for the construction of the numerical schemes in this work. As we will explain below, cf Lemma 1, the factor 1/*J* is important for the construction of $H({\text{div}}_{\Gamma })$‐conforming finite element spaces, that is, finite element spaces with continuous in‐plane normal components. We want to mention that in Rognes et al^17^ and Castro et al^50^ the authors already realized that the Piola mapping can be used on surfaces to construct appropriate finite element spaces for the approximation of the spaces $H({\text{div}}_{\Gamma })$ and $H({\mathrm{curl}}_{\Gamma })$ on Γ_*h*_. There, the according finite elements on the surface triangulation ${𝒯}_{h}$ are based on the Raviart‐Thomas and Brezzi‐Douglas‐Marini finite element families on the reference element $\hat{T}$, cf Girault and Raviart,^51^ Brezzi, Douglas and Marini,^52^ and Boffi, Brezzi and Fortin.^53^

### 3.2 A DG discretization for vector‐valued elliptic problems

**Figure 1:**
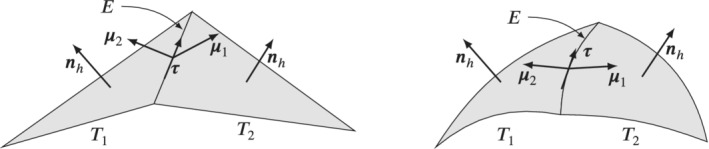
Two elements $T_{1},T_{2}\in {𝒯}_{h}$ with a common edge $E=\bar{T_{1}}\cap \bar{T_{2}}$ and the corresponding in‐plane normal vectors ***μ***_1_, ***μ***_2_, the common tangential vector ***τ***, and the oriented normal vector ***n***_*h*_. On the left with a linear geometry approximation, on the right side with a high order approximation.

Based on the findings from the last section we now motivate a new DG method for the approximation of problem (2). However, we want to mention that the discretization can also be adjusted to other elliptic problems like the Vector Laplacian without a mass term. To this end we need several operators motivated by their definitions on a flat surface, see Arnold et al.^54^ Let $T_{1},T_{2}\in {𝒯}_{h}$ be two arbitrary elements with common edge $E=\bar{T_{1}}\cap \bar{T_{2}}$. Let ***n***_*h*_ be the oriented unit normal vector on Γ_*h*_, and let **τ** be a uniquely oriented tangential vector on *E* such that *T*_1_,*T*_2_ are located on the left and on the right side, respectively, with respect to the direction of **τ**, see Figure 1. Using these vectors we define the *in‐plane* unit (outer) normal vectors

$$\begin{array}{ll} \mu_{1}:=n_{h}{|}_{T_{1}}\times \tau \;\text{and}\;\mu_{2}:=-n_{h}{|}_{T_{2}}\times \tau . & \; \end{array}$$

Let us stress here that ***n***_*h*_ will in general be discontinuous across element interfaces so that **μ**_1_ and **μ**_2_ will not be parallel.

Let *u* and σ be vector and matrix‐valued functions, respectively. We define the vector‐valued average and jump on *E* by

$$\begin{array}{ll} \{\{\sigma \mu \}\}:=\frac{\sigma {|}_{T_{1}}\mu_{1}-\sigma {|}_{T_{2}}\mu_{2}}{2}\;\text{and}\;[[u]]={\left[[u]\right]}_{\tau }\tau +{\left[[u]\right]}_{\mu }\bar{\mu } & \; \end{array}$$

with the scalar valued *normal* and *tangential jump* and the averaged in‐plane normal

(7) $$\begin{array}{ll} {\left[[u]\right]}_{\mu }:=u{|}_{T_{1}}\cdot \mu_{1}+u{|}_{T_{2}}\cdot \mu_{2},\;{\left[[u]\right]}_{\tau }:=(u{|}_{T_{1}}-u{|}_{T_{2}})\cdot \tau ,\;\bar{\mu }=\frac{1}{2}\mu_{1}+\frac{1}{2}\mu_{2}. & \; \end{array}$$

In the case of *E*⊂∂Γ≠∅ we set [[*u*]]_**μ**_=*u*·**μ** and the operators {{·}} and [[·]] are replaced by the identity. Based on the Piola mapping, see Equation (6), we define a finite element space of order *k* by

$$\begin{array}{ll} W_{h}^{k}:=\{v\in {\left[L^{2}(\Gamma_{h})\right]}^{3}:\forall T\in {𝒯}_{h}\;\exists û\in {\left[P^{k}(\hat{T})\right]}^{2}\;\text{s.t.}\;v{|}_{T}={𝒫}_{T}(û)\}. & \; \end{array}$$

Here, $P^{k}(\hat{T})$ is the scalar‐valued polynomial space of order *k* on $\hat{T}$. By construction, we have that

(8) $$\begin{array}{ll} u_{h}(x)\cdot n_{h}(x)=0\;\forall x\in \Gamma_{h},\;\forall u_{h}\in W_{h}^{k}, & \; \end{array}$$

thus discrete functions in $W_{h}^{k}$ are exactly in the tangent plane of Γ_*h*_. Assuming that *f*, originally defined on Γ, has a smooth extension on Γ_*h*_, we follow Arnold et al^54^ to define the DG method (based on an symmetric interior penalty formulation): Find $u_{h}\in W_{h}^{k}$ such that

(9a) $$\begin{array}{ll} a_{h}(u_{h},v_{h})+m_{h}(u_{h},v_{h})=\int_{\Gamma_{h}}f\cdot v_{h}ds=:f_{h}(v_{h})\;\forall v_{h}\in W_{h}^{k}, & \; \end{array}$$

where

(9b) $$\begin{array}{ll} a_{h}(u_{h},v_{h}) & :=\underset{T\in {𝒯}_{h}}{\sum }\int_{T}\varepsilon_{\Gamma }(u_{h}):\varepsilon_{\Gamma }(v_{h})dx+\underset{E\in {ℱ}_{h}}{\sum }\int_{E}\{\{-\varepsilon_{\Gamma }(u_{h})\}\}:[[v_{h}]]ds+\int_{E}\{\{-\varepsilon_{\Gamma }(v_{h})\}\}:[[u_{h}]]ds\\ & \;+\frac{\alpha k^{2}}{h}\int_{E}[[u_{h}]]:[[v_{h}]]ds. \end{array}$$

with the constant α chosen big enough, see Arnold et al,^54^ and $m_{h}(u_{h},v_{h})=\int_{\Gamma_{h}}Pu_{h}v_{h}ds$. Note that the projection *P* in the definition of the bilinear form *m*_*h*_(·,·) is redundant for functions in $W_{h}^{k}$. However, we will use *m*_*h*_(·,·) for nontangential functions as well later on.

Remark 3 In the case of nonhomogeneous Dirichlet or other types of boundary conditions one uses the standard techniques employed for DG methods on flat surfaces, see Arnold et al.^54^

Remark 4 The application of the Piola mapping in the definition of functions in $W_{h}^{k}$ results in exactly tangential fields. Furthermore, it results in a discretization that is—as the continuous problem—invariant under isometries, that is, if *u*_*h*_ solves (9b) on Γ_*h*_, $𝒫(u_{h})$ solves the corresponding problem on $\Gamma_{h}^{\prime }=\Phi (\Gamma_{h})$ if Φ is an isometry and 𝒫 the corresponding Piola mapping. We discuss this in more detail below in the numerical examples, cf Sections 4.1.3 and 4.3.1 below.

### 3.3 Discretization of the surface Stokes equations

In this section we focus on the construction of a numerical scheme to solve the Stokes problem on Γ, see Equation (4). Note that we assumed ∂Γ≠∅ and thus also ∂Γ_*h*_≠∅. As known from the literature, see for example Boffi, Brezzi, Fortin^53^ and Braess,^55^ discrete stability demands to find a compatible pair of the discrete velocity and pressure space. We aim to construct a method based on the works^41^, ^43^, ^56^ for the flat case. In the latter works, the discrete velocity space is chosen to be the H(div)‐conforming Brezzi‐Douglas‐Marini (BDM) space of order *k*_*u*_, and the pressure is approximated by discontinuous polynomials of order *k*_*u*_  − 1. These two spaces fulfill the property that the divergence of the velocity space is a subspace of the discrete pressure space. This ensures stability in the sense of Babuška‐Brezzi, see for example Reference 44, and leads to major benefits such as exactly divergence‐free discrete velocities, see also Section 3.5. Following the ideas of Rognes et al^17^ and Castro et al,^50^ we now map this method to the surface using the previously introduced Piola mapping, see Equation (6), to define an $H({\text{div}}_{\Gamma })$‐conforming velocity space on Γ_*h*_. Then for an arbitrary order *k* we set

(10) $$\begin{array}{ll} V_{h}^{k}:=\{u_{h}\in W_{h}^{k}:{\left[[u_{h}]\right]}_{\mu }=0\;\forall E\in {ℱ}_{h}\}=W_{h}^{k}\cap H_{0}({\text{div}}_{\Gamma }), & \; \end{array}$$

where $H_{0}({\text{div}}_{\Gamma })$ is the subspace of $H({\text{div}}_{\Gamma })$ with zero normal trace at the boundary ∂Γ. Regarding the implementation of $V_{h}^{k}$ note, that the Piola mapping not only helps to incorporate property (8), but can also be used to incorporate the zero normal jump. This is done by choosing the BDM basis on the reference element $\hat{T}$ and map it with the Piola transformation to the physical elements $T\in {𝒯}_{h}$. As the BDM basis functions are associated to the scalar normal moments on the edges of the reference element, the mapped functions are then associated to the according (in‐plane) normal moments on the edges of the mapped element *T*. This automatically results in a normal continuous function, that is, [[*u*]]_**μ**_=0 on all edges. A detailed discussion on this topic is given in Castro et al.^50^ Next we define the discrete pressure space as

$$\begin{array}{ll} Q_{h}^{k}:=\{q\in L^{2}(\Gamma_{h}):\forall T\in {𝒯}_{h}\;\exists \hat{q}\in P^{k}(\hat{T})\;\text{s.t.}\;q{|}_{T}=\hat{q}\circ \Phi_{T}^{-1}\}\cap L_{0}^{2}(\Gamma_{h}). & \; \end{array}$$

For a fixed velocity approximation order *k*_*u*_ the $H({\text{div}}_{\Gamma })$‐conforming DG method then read as: Find $(u_{h},p_{h})\in V_{h}^{k_{u}}\times Q_{h}^{k_{u}-1}$ such that

(11) $$\begin{array}{ll} \begin{array}{rll} a_{h}(u_{h},v_{h})+b_{h}(v_{h},p_{h}) & =f_{h}(v_{h}) & \;\forall v_{h}\in V_{h}^{k_{u}},\\ b_{h}(u_{h},q_{h}) & =0 & \;\forall q_{h}\in Q_{h}^{k_{u}-1}, \end{array} & \; \end{array}$$

with

$$\begin{array}{ll} b_{h}(u_{h},q_{h})=-\int_{\Gamma_{h}}{\text{div}}_{\Gamma }(u_{h})q_{h}dx. & \; \end{array}$$

Note that the appearing jumps in *a*_*h*_(*u*_*h*_,*v*_*h*_) now only read as the tangential jump since functions in $V_{h}^{k_{u}}$ are normal continuous. As a consequence we have the following lemma.

Lemma 1 *Let* $u_{h}\in V_{h}^{k_{u}}$ *such that* $b_{h}(u_{h},q_{h})=0\;\forall q_{h}\in Q_{h}^{k_{u}-1}$ *. Then u*_*h*_ *is exactly divergence‐free, that is,* ${\text{div}}_{\Gamma }(u_{h})=0$ *on* Γ_*h*_.

For each $T\in {𝒯}_{h}$ let ${û}_{h,T}\in P^{k_{u}}(\hat{T},R^{2})$ be such that $u_{h}={𝒫}_{T}({û}_{h,T})$. Applying the chain rule shows

(12) $$\begin{array}{ll} {\text{div}}_{\Gamma }(u_{h})=\frac{1}{J}\text{div}({û}_{h,T}) & \; \end{array}$$

where $\text{div}({û}_{h,T})$ reads as the divergence on the flat reference element $\hat{T}$. As ${û}_{h,T}\in P^{k_{u}}(\hat{T},R^{2})$ we have $\text{div}({û}_{h,T})\in P^{k_{u}-1}(\hat{T})$. Choosing ${\hat{q}}_{h,T}=\mathrm{sgn}(J)\text{div}({û}_{h,T})$ and $q_{h,T}={\hat{q}}_{h,T}\circ \Phi^{-1}$, we define the global function *q*_*h*_ as $q_{h}=q_{h}^{*}+c$ with $q_{h}^{*}{|}_{T}=q_{h,T}$ and the global constant c∈R such that $\int_{\Gamma_{h}}q_{h}=0$, thus $q_{h}\in Q_{h}^{k_{u}-1}$. Then we have with $b_{h}(u_{h},c)=-c\int_{\Gamma_{h}}{\text{div}}_{\Gamma }(u_{h})dx=-c\int_{\partial \Gamma_{h}}u_{h}\cdot \mu ds=0$ that there holds

$$\begin{array}{ll} 0=-b_{h}(u_{h},q_{h})=\int_{\Gamma_{h}}{\text{div}}_{\Gamma }(u_{h})q_{h}^{*}dx=\underset{T\in {𝒯}_{h}}{\sum }\int_{T}{\text{div}}_{\Gamma }(u_{h})q_{h,T}dx=\underset{T\in {𝒯}_{h}}{\sum }\int_{\hat{T}}\frac{1}{J}\mathrm{sgn}(J)\text{div}{\left({û}_{h,T}\right)}^{2}|J|dx=\underset{T\in {𝒯}_{h}}{\sum }\int_{\hat{T}}\text{div}{\left({û}_{h,T}\right)}^{2}dx & \; \end{array}$$

which shows that $\text{div}({û}_{h,T})=0$, thus we conclude by Equation (12).

From Lemma 1 we conclude that the solution *u*_*h*_ of (11) is exactly divergence‐free. Note that this statement is completely independent of the geometry approximation. This further leads to pressure robustness (see Sections 3.5 and 4.2) and shows that the function spaces $V_{h}^{k_{u}}$ and $Q_{h}^{k_{u}-1}$ are compatible, thus the stability proof of (11) follows the same steps as in the flat case, see References 42 and 43.

### 3.4 Discretization of the surface Navier‐Stokes equations

The discretization of the Navier‐Stokes problem (5) follows the ideas of References 42 and 43 which we briefly summarize in the following. We aim to use a semidiscrete method to decouple the discretization in space and time. This is then further combined with an (high order) operator splitting method to efficiently deal with the nonlinear convection term. For the latter one we use a standard upwinding scheme in space which guarantees energy stability. To this end let $w_{h},u_{h},v_{h}\in V_{h}^{k_{u}}$, then we define the form

(13) $$\begin{array}{ll} c_{h}(w_{h})(u_{h},v_{h})=\underset{T\in {𝒯}_{h}}{\sum }-\int_{T}u_{h}⊗w_{h}:\nabla v_{h}dx+\int_{\partial T}w_{h}\cdot \mu \;u_{h}^{\mathrm{up}}\cdot v_{h}ds, & \; \end{array}$$

where the upwinding value is chosen in upstream direction with respect to the convection velocity *w*_*h*_: Let *x*∈*∂T* and *T′* be the neighboring element, s.t. *x*∈*T*,*T′*, then we define

$$u_{h}^{\mathrm{up}}:=(u_{h}\cdot \mu_{T})\cdot \mu_{T}+(u_{h}^{\mathrm{up},\tau }\cdot \tau )\cdot \tau ,\;\text{where}\;u_{h}^{\mathrm{up},\tau }(x)=u_{h}^{\mathrm{up},\tau }{|}_{T}(x)\;\text{if}\;u_{h}\cdot \mu_{T}\geq 0\;\text{and}\;u_{h}^{\mathrm{up},\tau }(x)=u_{h}^{\mathrm{up},\tau }{|}_{T^{\prime }}(x)\;\text{otherwise.}$$

We notice that the upwinding only affects the discontinuous tangential component. This type of formulation is typically derived by applying partial integration in an element‐by‐element fashion and introducing *numerical fluxes*, cf for example Hesthaven and Warburton.^57^ Let us further note that the integration by parts formula for vector fields that are not exactly tangential involves an additional term including the mean curvature, cf Fries^38^ eq. (3).

In combination with the bilinear forms defined in the previous section and again assuming that the initial velocity *u*_0_ has a smooth extension onto Γ_*h*_, we derive the following spatially discrete differential‐algebraic equation (DAE) problem: find $u_{h}(t)\in V_{h}^{k_{u}},p_{h}(t)\in Q_{h}^{k_{u}-1}$ such that

(14a) $$\begin{array}{llll} m_{h}(\frac{\partial }{\partial t}u_{h},v_{h})+2\nu a_{h}(u_{h},v_{h})+b_{h}(v_{h},p_{h})+c_{h}(u_{h})(u_{h},v_{h}) & =f_{h}(v_{h})\; & & \forall v_{h}\in V_{h}^{k_{u}},t\in (0,T], \end{array}$$

(14b) $$\begin{array}{llll} \;b_{h}(u_{h},q_{h}) & =0\; & & \forall q_{h}\in Q_{h}^{k_{u}-1},t\in (0,T], \end{array}$$

(14c) $$\begin{array}{llll} \;m_{h}(u_{h},v_{h}) & =m_{h}(u_{0},v_{h})\; & & \forall v_{h}\in V_{h}^{k_{u}},t=0, \end{array}$$

where (14c) resembles the *L* ^2^‐projection of the initial velocity onto the discrete velocity space $V_{h}^{k_{u}}$. Now let ${\underline{u}}_{h}(t)$ and ${\underline{p}}_{h}(t)$ be the finite element coefficient vectors of the functions *u*_*h*_,*p*_*h*_, respectively, and let *t*_*i*_ with *i*=*i*,…,*N* where *t*_0_=0,*t*_*N*_=*T* be an equidistant mesh for [0,*T*] with time step size Δ*t*. To get the solution at time *t*_*i*+1_ we solve a step of the fully discrete lowest order implicit explicit (IMEX) splitting scheme

(15) $$\begin{array}{ll} \begin{array}{rl} M\frac{{\underline{u}}_{h}(t_{i+1})-{\underline{u}}_{h}(t_{i})}{\Delta t}(\frac{\partial }{\partial t}{\underline{u}}_{h})+2\nu A{\underline{u}}_{h}(t_{i+1})+B^{T}{\underline{p}}_{h}(t_{i+1}) & =f_{h}(v_{h})-C({\underline{u}}_{h}(t_{i})){\underline{u}}_{h}(t_{i}),\\ B{\underline{u}}_{h}(t_{i+1}) & =0. \end{array} & \; \end{array}$$

Here the matrices *M*,*A*,*B* are the corresponding matrices of the bilinear forms *m*_*h*_,*a*_*h*_ and *b*_*h*_ respectively, and $C({\underline{u}}_{h}(t_{i})){\underline{u}}_{h}(t_{i})$ reads as the evaluation of the convection trilinear form *c*_*h*_ with the wind *w*_*h*_=*u*_*h*_(*t*_*i*_). Above system shows that we treat the stiffness *A* and divergence constraint *B* implicitly which guarantees exactly divergence‐free velocity solutions at each point in time. The convection *C*, however, is treated explicitly. For more details regarding the efficiency and accuracy of above splitting methods for the Navier‐Stokes equations we refer to References 42 and 43 for the flat case. The simplest variant to generalize (15) to higher order order in time are IMEX schemes, cf the works by Ascher et al.^58^, ^59^ Below in the numerical examples we use a second‐order IMEX based on two compatible explicit and implicit Runge‐Kutta schemes of second order.

### 3.5 On the benefits of $H({\text{div}}_{\Gamma })$‐conformity

Additionally to the fact that the $H({\text{div}}_{\Gamma })$‐conforming space $V_{h}^{k_{u}}$ is tangential there are several advantages over other Stokes discretizations (see also the HDG Stokes discretization introduced in Section 4.2). Below, we focus on two important ones. For other aspects—which transfer directly from the flat case—such as the ability to reduce the set of basis functions, convection stability (beyond energy stability) and good dissipation properties we refer to the literature, for example References 42 43, and 60.

#### 3.5.1 Pressure robustness

As discussed in Section 3.3, Lemma 1 shows that solutions *u*_*h*_ of (11) are exactly divergence‐free independently of the geometry approximation. Further, the combination of the velocity space $V_{h}^{k_{u}}$ and the pressure space $Q_{h}^{k_{u}-1}$ allows to test Eq. (11) with exactly divergence‐free velocity test functions. This is a crucial advantage as it enables us to derive velocity error estimates that are independent of the pressure approximation (and the viscosity ν). This property is known in the literature as *pressure robustness* and was first introduced by Linke.^61^ In the following we briefly sketch the main idea (hence the occurring problem): let the r.h.s. *f* of the Stokes problem be decomposed as

$$\begin{array}{ll} f=\nabla_{\Gamma }\theta +\xi , & \; \end{array}$$

with $\theta \in H^{1}(\Gamma )/R$ and ξ∈[*L* ^2^(Γ)]^3^ such that ξ· ***n*** =0. Testing the continuous problem (4) with a test function $v\in V_{0}:=\{v\in H({\text{div}}_{\Gamma },\Gamma ):{\text{div}}_{\Gamma }(v)=0\}$ we see that $2\nu a(u,v)=\int_{\Gamma }\xi \cdot vdx$, hence the velocity is not steered by the gradient ∇_Γ_θ. If the same observation can be made in the discrete setting the method is called pressure robust and one can derive velocity error estimates that are independent of the pressure approximation (see Section 4.2 for a numerical observation of this phenomenon). A rigorous analysis of such (Helmholtz) decompositions on smooth manifolds is given in Reusken.^34^ For the above findings in the continuous setting it was crucial that one tests with exactly divergence‐free test functions *v*∈*V*_0_. Lemma 1 shows that the same can be done in the discrete setting which leads to pressure robustness of the discretization given by Equation (11). Note, that also standard methods can be made pressure robust, see References 62 to 65. Further, it also plays an important role in the Navier‐Stokes case.^66^, ^67^, ^68^, ^69^

#### 3.5.2 Energy stability

The standard DG upwind formulation of the convection bilinear form (13) allows to show the following stability result (up to geometry errors). Let ∂Γ_*in*_:={*x*∈∂Γ:*w*_*h*_·**μ**<0} then there holds

$$\begin{array}{ll} \frac{1}{2}\int_{\partial \Gamma_{in}}|w_{h}\cdot \mu |u_{h}^{2}ds+c_{h}(w_{h})(u_{h},u_{h})\geq 0\;\forall u_{h},w_{h}\in V_{h}^{k_{u}}\;\text{with}\;{\text{div}}_{\Gamma }(w_{h})=0. & \; \end{array}$$

Note that the crucial assumption here is that the transport velocity *w*_*h*_ is exactly (surface) divergence‐free. Considering the time discretization (15) of the Navier‐Stokes equations, the transport velocity was chosen as the discrete velocity of the old time step, that is, *w*_*h*_=*u*_*h*_(*t*_*i*_). Thus, as ${\text{div}}_{\Gamma }(u_{h}(t_{i}))=0$ (exactly) we can apply above stability estimate showing energy stability of the Navier‐Stokes discretization.

### 3.6 HDG formulations

Although the formulation of Section 3.2 fulfills property (8), the computational overhead introduced by the DG formulation is a major drawback. A well known technique to overcome this circumstance is to use HDG formulations and a static condensation strategy. The idea is to introduce additional unknowns on the skeleton ${ℱ}_{h}$ which circumvents the direct coupling of element (interior) unknowns with neighboring elements. We give two examples how this can be achieved and comment on further efficiency improvements. Below, we will use HDG discretizations for the numerical examples.

#### 3.6.1 An HDG method for the Vector Laplacian

Let Ψ_*E*_ be the unique polynomial map from the reference edge Ê=[0,1] to an edge $E\in {ℱ}_{h}$, with $E=\bar{T_{1}}\cap \bar{T_{2}},T_{1},T_{2}\in {𝒯}_{h}$ as before. We define the space of (discontinuous) piecewise polynomials on the skeleton as

$$\begin{array}{ll} \Lambda_{h}^{k}:=\{v\in L^{2}({ℱ}_{h}):\forall E\in {ℱ}_{h}\;\exists \hat{v}\in P^{k}(Ê)\;\text{s.t.}\;v{|}_{E}=\hat{v}\circ \psi_{E}^{-1}\}. & \; \end{array}$$

Here, $P^{k}(Ê)$ is the scalar‐valued polynomial space of order *k* on Ê. Then we define the following two vector‐valued functions

$$\begin{array}{ll} \lambda_{T_{1}}:=\lambda_{a}\tau +\lambda_{b}\mu_{1}\;\text{and}\;\lambda_{T_{2}}:=\lambda_{a}\tau +\lambda_{b}\mu_{2}\;\text{for}\;\lambda_{a},\lambda_{b}\in \Lambda_{h}^{k}, & \; \end{array}$$

where **τ** and **μ**_1_,**μ**_2_ are defined as above. Here λ_*a*_ is introduced to decouple the weak (in the DG sense) tangential continuity whereas λ_*b*_ is introduced for the weak normal continuity. Note, that $\lambda_{T_{1}}$ might not be equal to $\lambda_{T_{2}}$ as the in‐plane normal vector may be different. The HDG method then reads as: Find $u_{h},\lambda_{T}\in W_{h}^{k}\times (\Lambda_{h}^{k}\times \Lambda_{h}^{k})$ such that

$$\begin{array}{ll} a_{h}^{\mathrm{HDG}}((u_{h},\lambda_{T}),(v_{h},\theta_{T}))+m_{h}(u_{h},v_{h})=f_{h}(v_{h})\;\forall (v_{h},\theta_{T})\in W_{h}^{k}\times (\Lambda_{h}^{k}\times \Lambda_{h}^{k}), & \; \end{array}$$

where

$$\begin{array}{ll} a_{h}^{\mathrm{HDG}}((u_{h},\lambda_{T}),(v_{h},\theta_{T})):=\underset{T\in {𝒯}_{h}}{\sum } & \int_{T}\varepsilon_{\Gamma }(u_{h}):\varepsilon_{\Gamma }(v_{h})dx+\int_{\partial T}(\varepsilon_{\Gamma }(u_{h})\;\mu )\cdot (\theta_{T}-v_{h})ds\;\\ & +\int_{\partial T}(\varepsilon_{\Gamma }(v_{h})\;\mu )\cdot (\lambda_{T}-u_{h})ds+\frac{\alpha k^{2}}{h}\int_{\partial T}(\lambda_{T}-u_{h})\cdot (\theta_{T}-v_{h})ds.\; \end{array}$$

The HDG method has more unknowns compared to the DG version, but the element unknowns $u_{h}\in W_{h}^{k}$ depend only on the facet function λ_*T*_. Hence, in a linear system, we can form the Schur complement with respect to those unknowns which is known as *static condensation*. This can be done in an element‐by‐element fashion already during the setup of the linear systems. The Schur complement system is typically much smaller, especially for higher order elements (note that the facet unknowns scale with *k* whereas the element unknowns scale with *k* ^2^). An example for the impact of static condensation with HDG methods in comparison to DG methods will be given in Section 4.1.2.

#### 3.6.2 An $H({\text{div}}_{\Gamma })$‐conforming HDG method for the Stokes and the Navier‐Stokes equations

Similarly to the standard DG method of Section 3.2, we can also use a hybridization technique for the $H({\text{div}}_{\Gamma })$‐conforming DG methods of Sections 3.3 and 3.4. As normal continuity is already incorporated in the space $V_{h}^{k_{u}}$, we do not need the facet variable λ_*b*_. Hence the facet variable on the skeleton ${ℱ}_{h}$ reduces to the single valued quantity

$$\begin{array}{ll} \lambda_{T_{1}}=\lambda_{T_{2}}=\lambda_{a}\tau \;\text{with}\;\lambda_{a}\in \Lambda_{h}^{k_{u}}, & \; \end{array}$$

as the tangential vector **τ** is the same on both sides. With the definitions λ_*T*_(λ_*a*_,*u*_*h*_):=λ_*a*_ **τ**+(*u*_*h*_·**μ**)**μ** and θ_*T*_(θ_*a*_,*u*_*h*_):=θ_*a*_ **τ**+(*u*_*h*_·**μ**)**μ**, we can use the same HDG bilinear form $a_{h}^{\mathrm{HDG}}$ for the treatment of viscosity. For the convection operator we have to replace the upwind flux $u_{h}^{\text{up}}$, cf (13), to avoid communication between interior element unknowns in $V_{h}^{k_{u}}$. Hence, we make the following choice:

$$\begin{array}{ll} u_{h}^{\mathrm{HDG},\mathrm{up}}:=(u_{h}\cdot \mu )\mu +\left\{\begin{array}{cc} (u_{h}\cdot \tau )\tau \; & \text{if}\;w_{h}\cdot \mu >0,\\ \lambda_{a}\tau \; & \text{else}. \end{array}\right. & \; \end{array}$$

Further, to make sure that λ_*a*_ takes the value of the upwind neighbor also in the convection dominated regime we add a consistent stabilization (the “downwind glue”) as introduced in Egger and Schöberl^70^ and define

$$\begin{array}{llll} c_{h}^{\mathrm{HDG}}(w_{h})((u_{h},\lambda_{T}),(v_{h},\theta_{T})) & :=\underset{T\in {𝒯}_{h}}{\sum }-\int_{T}u_{h}⊗w_{h}:\nabla v_{h}dx+\int_{\partial T}w_{h}\cdot \mu \;u_{h}^{\mathrm{HDGup}}\cdot v_{h}ds\;\\ & \;+\int_{\partial T_{\text{out}}}w_{h}\cdot \mu \;(\lambda_{T}-(u_{h}\cdot \tau )\tau )\cdot \theta_{T}ds, & & \end{array}$$

where *∂T*_out_ denotes the outflow boundary of an element, that is, *∂T*_out_:=*∂T*∩{*w*_*h*_·**μ**>0}. Let us stress that in the convective limit ν→0, the HDG solution converges to the DG solution discussed above.

The semidiscretization of the new hybrid $H({\text{div}}_{\Gamma })$‐conforming DG method for the Navier‐Stokes equations then reads: Find $(u_{h},\lambda_{a},p_{h})\in V_{h}^{k_{u}}\times \Lambda_{h}^{k_{u}}\times Q_{h}^{k_{u}-1}$ such that

(16a) $$\begin{array}{llll} m_{h}(\frac{\partial }{\partial t}u_{h},v_{h})+2\nu a_{h}^{\mathrm{HDG}}((u_{h},\lambda_{T}),(v_{h},\theta_{T}))\;\; & \; & & \;\\ +c_{h}(u_{h})((u_{h},\lambda_{T}),(v_{h},\theta_{T}))+b_{h}(v_{h},p_{h}) & =f_{h}(v_{h})\; & & \;\forall v_{h},\theta_{a}\in V_{h}^{k_{u}}\times \Lambda_{h}^{k_{u}},t\in (0,T], \end{array}$$

(16b) $$\begin{array}{llll} \;b_{h}(u_{h},q_{h}) & =0\; & & \;\forall q_{h}\in Q_{h}^{k_{u}-1},t\in (0,T], \end{array}$$

(16c) $$\begin{array}{llll} \;m_{h}(u_{h},v_{h})-m_{h}(u_{0},v_{h}) & =0\; & & \;\forall v_{h}\in V_{h}^{k_{u}},t=0, \end{array}$$

where we implicitly made use of λ_*T*_=λ_*T*_(λ_*a*_,*u*_*h*_) and θ_*T*_=θ_*T*_(θ_*a*_,*v*_*h*_). Using this spatial discretization we can then again use the implicit‐explicit splitting scheme for the discretization of the Navier‐Stokes equations on Γ_*h*_, see Equation (15).

##### 3.6.2.1 Efficiency aspects and superconvergence

One advantage of HDG methods that we did not address so far is the ability to achieve superconvergence in diffusion dominated problems. If the (trace) unknowns on the skeleton are approximated with polynomials of order *k*, one can (for example) apply a local postprocessing strategy (see Cockburn et al^71^) to define a local elementwise approximation of order *k* + 1 which has order *k* + 2 accuracy in the *L* ^2^ norm. Alternatively, one can consider the previous HDG formulation and reduce the facet degree by one order while preserving the order of accuracy by introducing a slight modification in the formulation, cf Reference [43, sect. 2.2]. In the context of H(div)‐conforming methods, the reduction of the polynomial degree for the normal component requires a bit more care in order to preserve the beneficial properties of H(div)‐conforming methods, cf References 45 and 46.

## 4 NUMERICAL EXAMPLES

In this section we consider several numerical examples. We start with a comparison of [*H* ^1^(Γ)]^3^‐conforming and the previously introduced nonconforming FME for the Vector Laplacian for smooth and piecewise smooth manifolds in Section 4.1. In the subsequent Section 4.2, we consider and compare the nonconforming methods for a surface Stokes problem. In the remainder we fix the method to the $H({\text{div}}_{\Gamma })$‐conforming HDG method and consider several surface versions of a well‐known benchmark problem in 2D in Section 4.3 and the Kelvin‐Helmholtz instability problem on different rotationally symmetric surfaces in Section 4.4. Finally, in Section 4.5 we consider a self‐organisation process on the Stanford bunny geometry. All numerical examples were implemented within the finite element library Netgen/NGSolve, see References 72 and 73 and www.ngsolve.org. The data, that is, time series, mesh refinement series, etc. as well as scripts for reproduction for all numerical examples can be found at DOI:10.5281/zenodo.3406173.

### 4.1 Vector Laplacian

First, we consider two Vector Laplace problems with different discretizations, compare and discuss them. In addition to those discretizations introduced before in Section 3.2, we consider two [*H* ^1^(Γ_*h*_)]^3^‐conforming discretizations from the literature that we briefly summarize in Section 4.1.1 below and which we denote as H1‐L (weak enforcement of tangential condition through Lagrangian multipliers) and H1‐P (weak enforcement of tangential condition through penalties). We introduce labels for the aforementioned methods. The method in (9) will be denoted as DG whereas the hybrid version of it is denoted as HDG. For the $H(\text{div},\Gamma_{h})$‐conforming discretization, that is, using (9) with $W_{h}^{k}$ replaced by $V_{h}^{k}$, cf (10), we use the label Hdiv‐DG and denote the HDG version as Hdiv‐HDG. For the numerical computations we only consider the HDG versions. Note however, that the differences between DG and HDG are primarily in a computational aspect, see also the explanations in Section 3.6. Hence, the DG versions are only considered for a comparison of this observation.

#### 4.1.1 *H* ^1^‐conforming discretizations for the Vector Laplacian

In the literature mostly [*H* ^1^(Γ_*h*_)]^3^‐conforming surface FEM discretizations for vector‐valued problems are considered by either applying Lagrangian multipliers for the tangential constraint or using a penalty formulation, see for example References 23 to 26 37, and 38. As we will use two instances of these methods for comparison to the methods discussed in Section 3, we briefly state these two formulations and discuss the choices involved. The methods are defined as follows: Find $(u_{h},\lambda_{h})\in {\left[S_{h}^{k}\right]}^{3}\times S_{h}^{k_{l}}$ s.t. $\forall (v_{h},\mu_{h})\in {\left[S_{h}^{k}\right]}^{3}\times S_{h}^{k_{l}}$:

(17a) $$\begin{array}{ll} (a_{h}+m_{h})(u_{h},v_{h})+\int_{\Gamma_{h}}(v_{h}\cdot n_{h})\lambda_{h}ds+\int_{\Gamma_{h}}(u_{h}\cdot n_{h})\mu_{h}ds & =f_{h}(v_{h}).\; \end{array}$$

Find $u_{h}\in {\left[S_{h}^{k}\right]}^{3}$ s.t. $\forall v_{h}\in {\left[S_{h}^{k}\right]}^{3}$:

(17b) $$\begin{array}{ll} (a_{h}+m_{h})(u_{h},v_{h})+\int_{\Gamma_{h}}\rho (u_{h}\cdot n_{h})(v_{h}\cdot n_{h})ds=f_{h}(v_{h}). & \; \end{array}$$

The [*H* ^1^(Γ_*h*_)]^3^‐conforming method H1‐L in (17a) uses Langrange multipliers to enforce the tangential constraint whereas (17b) enforces the tangential constraint through penalization with a penalty parameter ρ (H1‐P). For H1‐L the choice of the Lagrange multiplier space is *k*_*l*_=*k* as in Hansbo et al^23^ or Jankuhn and Reusken^25^ (in a TraceFEM context). We note that in Fries^38^ an *h* ^−6^ scaling of the condition number for Navier‐Stokes problems has been observed for *k*_*l*_=*k* and *k*_*l*_=*k*−1 has been used instead. To drive the normal component sufficiently small in the H1‐P method, we make the simple choice ρ=10·*h* ^−(*k*+1)^ and accept the severe ill‐conditioning of arising linear systems here. In Hansbo et al^23^ and Jankuhn and Reusken^25^ choosing such a large penalty is circumvented by replacing *ε*_Γ_(*u*_*h*_) (and *ε*_Γ_(*v*_*h*_)) by *ε*_Γ_(*Pu*_*h*_) (and *ε*_Γ_(*Pv*_*h*_)) which is a consistent manipulation. Thereby the normal and tangential parts of the discrete solution decouple and a *soft penalty* with a penalty parameter ρ∼*h* ^−2^ suffices.

Both choices taken here, *k*_*l*_=*k* for H1‐L and ρ=10·*h* ^−(*k*+1)^ for H1‐P will eventually result in severe ill‐conditioning of arising linear systems. However, for the test cases under consideration with H1‐L and H1‐P we used sparse direct solvers and obtained results of sufficient meaningfulness to serve as candidates for comparison.

#### 4.1.2 Vector Laplacian on the sphere

**Figure 2:**
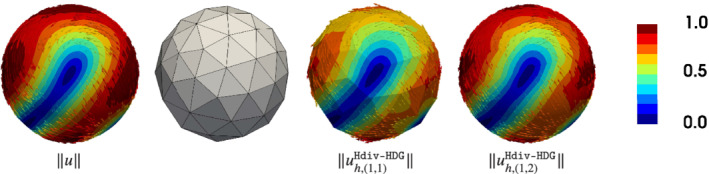
Exact and discrete geometry and solution for the Vector Laplacian in Section 4.1.2. Coloring corresponds to velocity magnitude. From left to right: exact geometry and solution, (uncurved) computational mesh on coarsest level *L*=0, mesh and discrete solution for Hdiv‐HDG with *k*=1, *k*_*g*_=1, and discrete solution for Hdiv‐HDG with *k*=1, *k*_*g*_=2 [Color figure can be viewed at wileyonlinelibrary.com]

We consider the Vector Laplace problem with the (extended) exact solution *u* ^*e*^=(−*z* ^2^,*y*,*x*)^*T*^ on the unit sphere Γ=*S*_1_(0), that is, we choose the r.h.s. *f* so that *u*=*u* ^*e*^|_Γ_ solves (2). For the geometry approximation we considered two choices, *k*_*g*_=*k* and *k*_*g*_=*k*+1 and for α in the SIP formulations we take α=10. On five successively and uniformly refined meshes we evaluate *L* ^2^‐ and *H* ^1^‐errors. The initial mesh (mesh level *L*=0) consists of 124 triangles, see Figure 2.

##### 4.1.2.1 Comparison of methods

**Figure 3:**
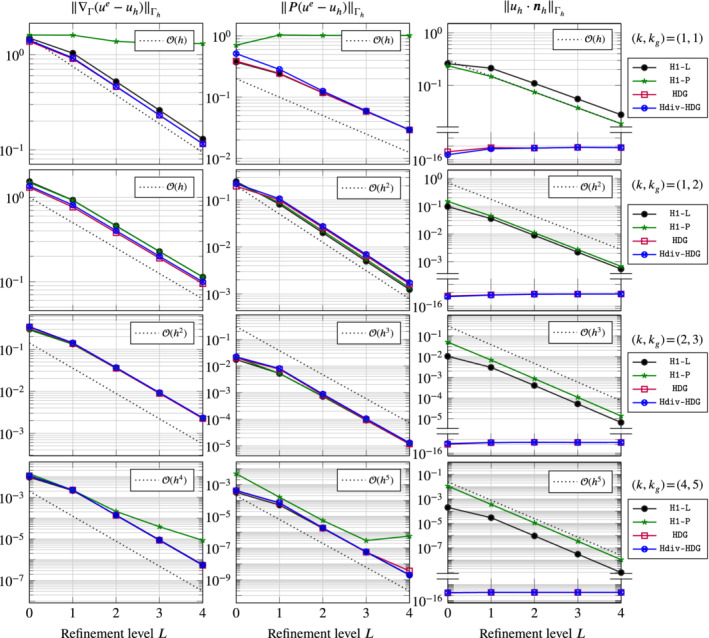
Errors for Vector Laplacian on the sphere for four different discretization methods on five successively refined meshes (uniform refinements) for different discretization and geometry orders [Color figure can be viewed at wileyonlinelibrary.com]

In Figure 3 we display the *H* ^1^‐seminorm, the *L* ^2^‐norm of the tangential and the *L* ^2^‐norm of the normal component of the error *u* ^*e*^−*u*_*h*_ on Γ_*h*_. Different configurations are considered.

In the first row we set geometry and FE approximation order to one, *k*_*g*_=*k*=1. First of all, we observe that the normal component is exactly zero only for the (H)DG methods. This is in agreement with our expectations as the *H* ^1^‐conforming methods only implement the tangential constraint weakly whereas the (H)DG methods fulfill the constraint by construction. Further, we notice that the convergence rates of all methods are suboptimal in all norms except for the *H* ^1^‐norm. For the *H* ^1^‐norm all methods are converging optimally with order one except for H1‐P which is not converging at all. This can be explained by the fact that the geometry approximation is only of first order so that the approximation of the normal is only piecewise constant. Elements surrounding the same vertex will have different normals and the penalty diminishes all corresponding normal components at the same time which results in a severe case of locking. In the Lagrange multiplier formulation H1‐L the constraint involves an averaging over a vertex patch which circumvents this locking effect. However, for all methods we observe that the low‐order approximation of the geometry results in a loss of accuracy by one order in the *L* ^2^ norms. This need for a higher order accurate geometry approximation is not surprising and has already been discussed in, for example, Hansbo et al^23^ and Jankuhn and Reusken.^25^ In Figure 2 a comparison of the pictures on the right illustrates the difference between *k*_*g*_=1 and *k*_*g*_=2 for *k*=1. In the rows two to four of Figure 3 we fix *k*_*g*_=*k* + 1, that is, a superparametric geometry approximation, and choose *k*∈{1,2,4}. For all methods we now observe optimal order of convergence, that is, $𝒪(h^{k})$ in the *H* ^1^‐seminorms and $𝒪(h^{k+1})$ in the *L* ^2^‐norms. For *k*=4, the results of H1‐P are not behaving optimally on the finest meshes. This is probably due to the ill‐conditioning stemming from the penalty parameter of magnitude 10*h* ^−5^. Let us further mention that we tried (for all methods) *k*_*g*_=*k* and obtained essentially the same results, that is, in this example it seems that the superparametric approximation is not necessary as long as *k*>1.

Overall, we can conclude that all methods perform similarly well for this example. The (H)DG methods are obviously—by construction—perfect in approximating the tangential constraint, but for the other error measures there is no significant difference.

##### 4.1.2.2 Computational aspects

In this paragraph we want to discuss and compare computational aspects of the different discretizaton methods under consideration for the Vector Laplacian. Starting with the *H* ^1^‐conforming methods, one immediately notices that the advantage of these methods is their simplicity. Lagrangian finite elements are available in most finite element packages and hence, a realization of these methods is comparably simple. Then again, the two‐dimensional vector field is approximated with a three‐dimensional vector field which can be seen as undesirable in terms of the computational overhead. To overcome this issue we introduced tangential finite elements. These however came at the price of abandoning *H* ^1^‐conformity which requires the implementation of weak continuity (at least in tangential direction) through the discrete variational formulation. This approach results in DG methods which come at the disadvantage of introducing more unknowns and more couplings. To alleviate these costs we also discussed the use of hybrid versions of DG methods. In Table 1 we compare the six different methods introduced before for polynomial orders 1 to 5 on the finest mesh *L*=4 of the previous example (Vector Laplacian on the sphere). While the *H* ^1^‐conforming and the HDG methods are exactly those investigated in the previous paragraph, the methods DG and Hdiv‐DG are only considered with respect to their computational costs, here. For the HDG methods and the *H* ^1^‐conforming methods we apply static condensation in an element‐by‐element fashion. As measures for the computational costs we take the number of degrees of freedom (dof), the number of global dof that remain in the Schur complement after static condensation (gdof) and the number of nonzero entries (nze) in the Schur complement.

**Table 1:** Comparison of different computational quantities (dof: degrees of freedom, gdof: global degrees of freedom that remain after static condensation (if applicable), nze: nonzero entries in system (Schur complement) matrix) for the different schemes on finest level

		dof						gdof						nze				
*k*		1	2	3	4	5		1	2	3	4	5		1	2	3	4	5
DG		193.5K	387.1K	645.1K	967.7K	1.4M		193.5K	387.1K	645.1K	967.7K	1.4M		4.6M	18.6M	51.6M	116.1M	227.6M
HDG		387.1K	677.4K	1M	1.5M	1.9M		193.5K	290.3K	387.1K	**483.8K**	**580.6K**		3.9M	8.7M	**15.5M**	**24.2M**	**34.8M**
Hdiv‐DG		96.8K	241.9K	451.6K	**725.8K**	**1.1M**		96.8K	241.9K	451.6K	725.8K	1.1M		2.5M	12M	36.9M	88.3M	180.6M
Hdiv‐HDG		193.5K	387.1K	645.1K	967.7K	1.4M		193.5K	290.3K	387.1K	**483.8K**	**580.6K**		3.9M	8.7M	**15.5M**	**24.2M**	**34.8M**
H1‐L		64.5K	258.1K	580.6K	1M	1.6M		64.5K	258.1K	451.6K	645.1K	838.7K		1.7M	11.1M	29.7M	48.1M	88.5M
H1‐P		**48.4K**	**193.5K**	**435.5K**	774.2K	1.2M		**48.4K**	**193.5K**	**338.7K**	**483.8K**	629K		**1M**	**6.7M**	16.7M	31.1M	49.8M

*Note*: Numbers in bold indicate that the corresponding method yields the best values in the current column.

Let us first take a look at the dof measure. Here, the H1‐P method has the smallest number of dof in the low‐order case *k*≤3. The additional dof for approximating a three‐dimensional vector field are still less than those obtained from approximating a two‐dimensional vector field with discontinuous piecewise polynomials. Only for *k*≥4 the Hdiv‐DG method with normal‐continuity results in less dof. When normal‐continuity is also broken up, that is, when going to DG it requires at least order *k*≥6 to beat H1‐P in terms of unknowns. When further going to HDG methods there is obviously no advantage over DG methods in terms of dof as only additional unknowns are introduced. As we can apply static condensation with HDG methods it is worth taking a look at those dof that remain in the Schur complement. The number of gdof can be reduced for DG for all polynomial degrees whereas the step from Hdiv‐DG to Hdiv‐HDG pays off in terms of gdof for *k*≥3. We observe that gdof is the same for HDG and Hdiv‐HDG. When considering nze the picture shifts even further towards HDG and Hdiv‐HDG. For *k*=2 already the methods HDG and Hdiv‐HDG outperform the other two DG methods and for *k*≥3 the method generates even less unknowns than the *H* ^1^‐conforming methods. We conclude that the hybrid formulations with tangential fields are beneficial not only because of their additional structure properties, but also computationally advantageous when going for higher order discretizations.

Remark 5 (HDG superconvergence) Let us note that we did not consider further tweaks of the HDG methods related to the aspect of superconvergence, cf the paragraph on efficiency aspects and superconvergence in Section 3.6. With a corresponding modification the polynomial degree on the facets can be reduced by one order while keeping the same order of accuracy (for diffusion dominated problems), that is, the costs in terms of gdof and nze for a method with accuracy *k* (in the *H* ^1^‐norm) are the same of those HDG methods without this modification with order *k*−1. In the lowest order case we obtatin gdof(HDG)=gdof(Hdiv‐HDG)=96.8K  and nze(HDG)=nze(Hdiv‐HDG)=967.7K  and hence, the HDG schemes are level with H1‐P in terms of gdof, but already more efficient in terms of nze.

#### 4.1.3 Vector Laplacian in the plane and on a house of cards

**Figure 4:**
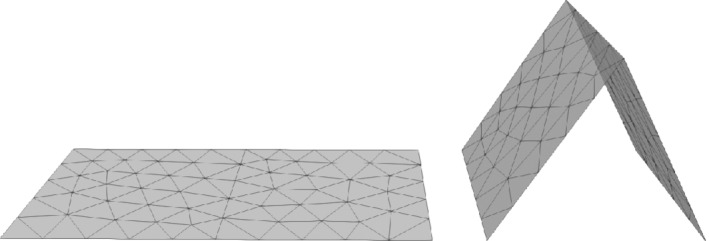
Geometries $\hat{\Gamma }$ (left) and Γ (right) and coarsest mesh (*L*=0) for the examples in Section 4.1.3

In this example we consider two configurations. First, we consider a flat domain case with $\hat{\Gamma }:=(0,2)\times (0,1)$ where we pose the Vector‐Laplacian problem (2) and replace (2b) with inhomogeneous Dirichlet boundary conditions *u*=*g* on ∂Γ. As a second example we fold the geometry in the middle and lift it up to a house of two cards:

$$\Gamma =\Phi_{H}(\hat{\Gamma })\;\text{with}\;\Phi_{H}(\hat{x},ŷ)=\left\{\begin{array}{ll} \left(\hat{x}\cdot W,ŷ,H\cdot \hat{x}\right) & \text{if}\;\hat{x}\leq 1,\\ \left(\hat{x}\cdot W,ŷ,H\cdot (2-\hat{x})\right) & \text{if}\;\hat{x}>1, \end{array}\right.$$

where *H* is the height and *W* is the width of one card with *H* ^2^+*W* ^2^=1 so that $F_{H}:=\Phi_{H}^{\prime }\in R^{3\times 2}$ is a length‐preserving map with $F_{H}^{T}F_{H}=I\in R^{2\times 2}$, cf Figure 4 for a sketch. We define with ${\hat{\tau }}_{1}=e_{1}=(1,0)$ and ${\hat{\tau }}_{2}=e_{2}=(0,1)$ the tangent unit vectors of $\hat{\Gamma }$. Then, $\tau_{i}=F_{H}\;{\hat{\tau }}_{i},i=1,2$ are the tangent unit vectors for Γ a.e., that is, we can interpret *F*_*H*_—and due to *J*=1 also the Piola transformation—as the operator that realizes a basis transformation from one tangent space to another.

On $\hat{\Gamma }$ we pose the Vector Laplacian with r.h.s. $\hat{f}$ and boundary data ĝ such that the exact solution to (2) on $\hat{\Gamma }$ is

$$û(\hat{x},ŷ)=(\sin(\pi \hat{x})+\cos(\pi ŷ))\;{\hat{\tau }}_{1}+(\cos(\pi \hat{x})+\sin(\pi ŷ))\;{\hat{\tau }}_{2}.$$

For the same problem on Γ (with properly transformed data $f=F_{H}\;\hat{f}\circ \Phi_{H}^{-1}$, $g=F_{H}\;ĝ\circ \Phi_{H}^{-1}$) the (extended) solution is simply

(18) $$u(x,y,z)=F_{H}\;û\circ \Phi_{H}^{-1}=(\sin(\pi \hat{x})+\cos(\pi ŷ))\;\tau_{1}+(\cos(\pi \hat{x})+\sin(\pi ŷ))\;\tau_{2}\;\text{with}\;(\hat{x},ŷ)=\Phi_{H}^{-1}(x,y,z)=(x/W,y).$$

Next, we discuss how this characterization of the solution translates to the discrete level. We note that Φ_*H*_ is piecewise affine, so that the Jacobian is piecewise constant. Let ${û}_{h}$ be the discrete solution to the Vector Laplace problem on $\hat{\Gamma }$ and $u_{h}=𝒫{û}_{h}$. Then, we can expand

$${û}_{h}=\overset{2}{\underset{i=1}{\sum }}{û}_{h,i}{\hat{\tau }}_{i},u_{h}:=\overset{=1}{\overbrace{J^{-1}}}F_{H}{û}_{h}\circ \Phi_{H}^{-1}=\underset{i}{\sum }\overset{=:u_{h,i}}{\overbrace{{û}_{h,i}\circ \Phi_{H}^{-1}}}\overset{=\tau_{i}}{\overbrace{F_{H}{\hat{\tau }}_{i}}}=\underset{i}{\sum }u_{h,i}\tau_{i}.$$

and obtain the following relation for the surface gradients:

$$\nabla_{\hat{\Gamma }}{û}_{h}=\nabla {û}_{h}=\overset{2}{\underset{i,j=1}{\sum }}\frac{\partial {û}_{h,i}}{\partial {\hat{\tau }}_{j}}{\hat{\tau }}_{i}⊗{\hat{\tau }}_{j},\;\nabla_{\Gamma }u_{h}=\underset{i,j}{\sum }\frac{\partial u_{h,i}}{\partial \tau_{j}}\tau_{i}⊗\tau_{j}=\underset{i,j}{\sum }\frac{\partial {û}_{h,i}}{\partial {\hat{\tau }}_{j}}\circ \Phi_{H}^{-1}\;\tau_{i}⊗\tau_{j}=F_{H}\overset{\in R^{2\times 2}}{\overbrace{\nabla {û}_{h}}}F_{H}^{T}\in R^{3\times 3}.$$

One may ask, if after applying the Piola transformation also on the test function, *u*_*h*_ solves the discrete Vector Laplace problem on Γ for the DG methods. One can easily check that this is true for the DG methods discussed here as there holds for instance

$$\nabla_{\Gamma }u_{h}:\nabla_{\Gamma }v_{h}=(F_{H}\nabla {û}_{h}F_{H}^{T}):(F_{H}\nabla {\hat{v}}_{h}F_{H}^{T})=(\underset{=I\in R^{2}}{\underbrace{F_{H}^{T}F_{H}}}\nabla {û}_{h}\underset{=I\in R^{2}}{\underbrace{F_{H}^{T}F_{H}}}):\nabla {\hat{v}}_{h}=\nabla {û}_{h}:\nabla {\hat{v}}_{h}.$$

Hence, *u*_*h*_ solves the discrete Vector Laplace problem on Γ for the DG discretizations if ${û}_{h}$ solves the discrete problem on $\hat{\Gamma }$. For the *H* ^1^‐conforming methods this does not apply as—for ${ŵ}_{h}$ being the discrete *H* ^1^‐conforming solution to the Vector Laplacian on $\hat{\Gamma }$—the mapped function $w_{h}=𝒫{ŵ}_{h}$ will not be in ${\left[S_{h}^{k}\right]}^{3}$ for *H*>0. By construction ${ŵ}_{h}$ is a continuous (2D) vector field s.t. after the transformation with Φ_*H*_, which has a discontinuous Jacobian, *w*_*h*_ is discontinuous and hence not included in ${\left[S_{h}^{k}\right]}^{3}$.

**Figure 5:**
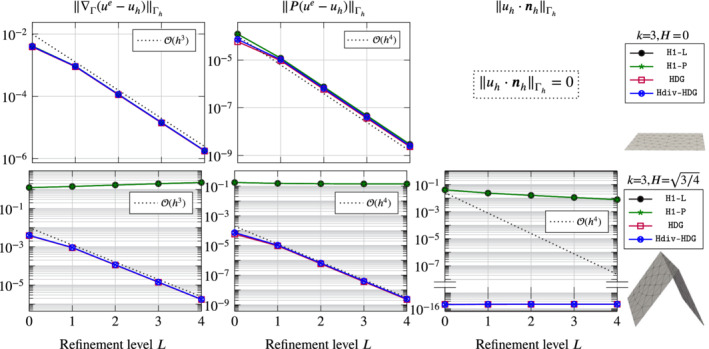
Error behavior for Vector Laplace problem on the flat (top row) and the house of cards geometry (bottom row) from Section 4.1.3 for four different discretization methods on five successively refined meshes (uniform refinements) for fixed discretization order *k*=3 and an exact geometry approximation [Color figure can be viewed at wileyonlinelibrary.com]

To illustrate this effect we consider the aforementioned methods with *k*=3 and $H\in \{0,\sqrt{3/4}\}$. In Figure 5 we display the error behavior for the two situations on five consecutive meshes, starting from the mesh displayed in Figure 4. Note that the geometry is piecewise planar, so that Γ_*h*_=Γ. For $\hat{\Gamma }$ we observe that all considered methods behave essentially the same. The deviation between the errors is only marginal. All methods yield an exactly tangential field *u*_*h*_· ***n***_*h*_=0 as the equations for the *z*‐component of the *H* ^1^‐conforming method completely separates from those in *x* and *y*‐direction. When going to Γ we observe that the *H* ^1^‐conforming methods fail to converge. This is easily explained by the fact that the solution is discontinuous when represented in the embedding space $R^{3}$. Considering the representation of the solution (18), we notice that the Piola mapping incorporates the tangential field automatically and we observe that the convergence behaviour of the DG methods stay the same when going from $\hat{\Gamma }$ to Γ. Actually, the numbers used in the plots are identical (up to round‐off errors).

### 4.2 Stokes

Whereas the last section demonstrated the accuracy and advantages of the (H)DG methods introduced in this work for second order elliptic problems on surfaces, we now aim to solve the stationary incompressible Stokes equations, see problem 4. Similarly as in the last section we define a Stokes problem on the flat reference domain $\hat{\Gamma }=(0,1)\times (0,1)$ and map it isometrically onto a smooth manifold given by half of the surface of an open cylinder with radius 1/π

$$\begin{array}{ll} \Gamma =\Phi_{1/\pi }(\hat{\Gamma })\;\text{with}\;\Phi_{1/\pi }(\hat{x},ŷ)=(\hat{x},\sin((ŷ-1/2)\pi )/\pi +1/\pi ,\cos((ŷ-1/2)\pi )/\pi ). & \; \end{array}$$

**Figure 6:**
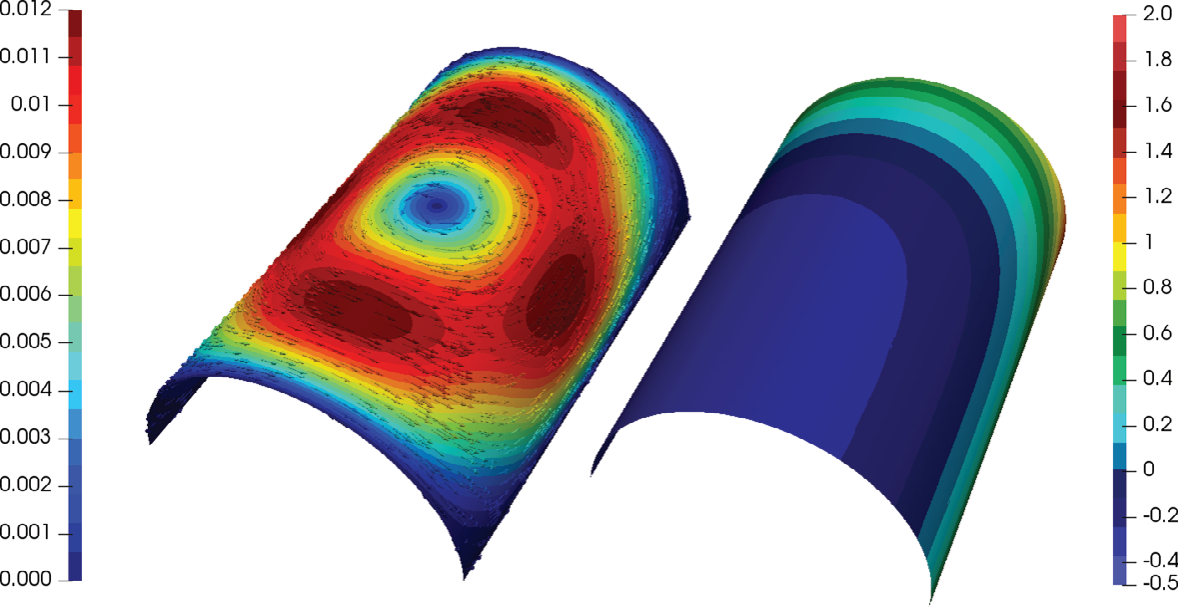
Absolute value |*u*| (left) of the exact velocity and the exact pressure *p* (right) given by (19) [Color figure can be viewed at wileyonlinelibrary.com]

Again, Φ_1/π_ preserves the length, that is, $\sqrt{\det(F_{1/\pi }^{T}F_{1/\pi })}=1$ with *F*_1/π_:=∇Φ_1/π_. For a fixed viscosity ν, the reference solutions are given by

$$\begin{array}{ll} û(\hat{x},ŷ)=-\frac{\partial \xi }{\partial ŷ}\cdot e_{1}+\frac{\partial \xi }{\partial \hat{x}}\cdot e_{2}\;\text{and}\;\hat{p}(\hat{x},ŷ)={\hat{x}}^{5}+{ŷ}^{5}-1/3 & \; \end{array}$$

with the scalar potential $\xi ={\hat{x}}^{2}{\left(1-\hat{x}\right)}^{2}{ŷ}^{2}{\left(1-ŷ\right)}^{2}$, and *e*_1_,*e*_2_ as before. We set the corresponding right hand side to $\hat{f}=-2\nu \varepsilon (û)+\nabla \hat{p}$. Defining the tangential vectors on Γ by **τ**_*i*_=*F*_1/π_ *e*_*i*_,*i*=1,2, the exact solutions are given by (see Figure 6)

(19) $$\begin{array}{ll} u(x,y,z)=-\frac{\partial \xi }{\partial ŷ}\cdot \tau_{1}+\frac{\partial \xi }{\partial \hat{x}}\cdot \tau_{2}\;\text{and}\;p(x,y,z)={\hat{x}}^{5}+{ŷ}^{5}-1/3, & \; \end{array}$$

with $\left(\hat{x},ŷ)=\Phi_{1/\pi }^{-1}(x,y,z)=(x,\arcsin(\pi y-1)/\pi +1/2\right)$, and the right hand side

(20) $$\begin{array}{ll} f(x,y,z)=f{\left(\hat{x},ŷ\right)}_{1}\cdot \tau_{1}+f{\left(\hat{x},ŷ\right)}_{2}\cdot \tau_{2}. & \; \end{array}$$

In the following we compare two different discretizations. The first one is the $H({\text{div}}_{\Gamma })$‐conforming HDG discretization, see Sections 3.6.1 and 3.3. The second one is based on the HDG formulation for the Vector Laplacian of Section 3.6.2. For the divergence constraint we now further introduce the bilinear form

$$\begin{array}{ll} b_{h}^{\mathrm{HDG}}((u_{h},\lambda_{T}),q_{h}):=-\underset{T}{\sum }\int_{T}{\text{div}}_{\Gamma }(u_{h})q_{h}dx+\int_{\partial T}(u-\lambda_{T})\cdot \mu \;q_{h}ds\;\forall u_{h},\lambda_{T}\in W_{h}^{k_{u}}\times (\Lambda_{h}^{k_{u}}\times \Lambda_{h}^{k_{u}}),q_{h}\in Q_{h}^{k_{u}-1}. & \; \end{array}$$

For a fixed velocity approximation order *k*_*u*_ the HDG Stokes discretization then read as: Find $u_{h},\lambda_{T}\in W_{h}^{k_{u}}\times (\Lambda_{h}^{k_{u}}\times \Lambda_{h}^{k_{u}}),p_{h}\in Q_{h}^{k_{u}-1}$ such that

(21) $$\begin{array}{ll} \begin{array}{rll} 2\nu a_{h}^{\mathrm{HDG}}((u_{h},\lambda_{T}),(v_{h},\theta_{T}))+b_{h}^{\mathrm{HDG}}((v_{h},\theta_{T}),p_{h}) & =f_{h}(v_{h}) & \;\forall (v_{h},\theta_{T})\in W_{h}^{k_{u}}\times (\Lambda_{h}^{k_{u}}\times \Lambda_{h}^{k_{u}})\\ b_{h}^{\mathrm{HDG}}((u_{h},\lambda_{T}),q_{h}) & =0 & \;\forall q_{h}\in Q_{h}^{k_{u}-1}. \end{array} & \; \end{array}$$

**Figure 7:**
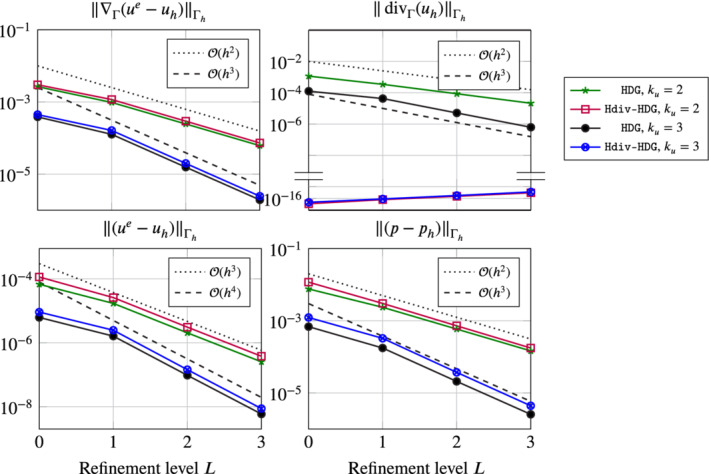
Error behavior for the Stokes problem of section 4.2 for the $H({\text{div}}_{\Gamma })$‐conforming HDG method and the HDG method given by (21) on four successively refined meshes (uniform refinements) for a fixed viscosity *ν*=1, two polynomial orders *k*_*u*_=2,3, and an geometry approximation order *k*_*g*_=*k*_*u*_+1 [Color figure can be viewed at wileyonlinelibrary.com]

In Figure 7 the error behavior for the above Stokes problem (with right‐hand side (20)) is given for both discussed discretizations with a fixed viscosity ν=1 and different polynomial orders *k*_*u*_=2,3. As before we use the labels HDG and Hdiv‐HDG. For α in the SIP stabilization we take 10. Note, that in contrast to the previous section we used the geometry approximation order *k*_*g*_=*k*_*u*_+2. A numerical investigation showed that we have to apply this enhanced geometry approximation (only needed for this particular example) due to the ill‐conditioned inverse of the sine function at 1 and −1 (needed for the calculation of the right‐hand side). The first, third, and fourth plot show the *H* ^1^‐seminorms error and the *L* ^2^‐norm error of the velocity and the *L* ^2^‐norm error of the pressure for both methods where we used an initial triangulation with 100 elements and three refinement levels. As we can see, all errors convergence with optimal order and the accuracy of both methods is approximately the same. The second plot shows the *L* ^2^‐norm of the surface divergence. As proven by Lemma 1 the solution of the $H({\text{div}}_{\Gamma })$‐conforming method is exactly divergence‐free, whereas the standard HDG method is only weakly divergence‐free. However the divergence error of the HDG method stills shows the optimal convergence order.

To emphasize the importance of exactly divergence‐free velocity solutions we now focus on pressure robustness. Beside the observations discussed in Section 3.5 we want to discuss pressure robustness with respect to the arising error estimates. A standard a priori error estimate of inf‐sup stable Stokes discretizations usually reads as

$$\begin{array}{ll} \|u-u_{h}{\|}_{H^{1},h}\leq c(\underset{v_{h}\in V_{h}^{k_{u}}}{\inf}\|u-v_{h}{\|}_{H^{1},h}+\frac{1}{\nu }\underset{q_{h}\in Q_{h}^{k_{u}-1}}{\inf}\|p-q_{h}{\|}_{L^{2}}), & \; \end{array}$$

where $\|\cdot {\|}_{H^{1},h}$ is an appropriate (H)DG‐version of an *H* ^1^‐norm, and *c* is a constant independent on the mesh size *h* and the viscosity ν. Above estimate shows that the velocity error may depend on the best approximation of the pressure including the factor 1/ν, hence the velocity error can blow up in the case of vanishing viscosity ν≪1. As discussed in the literature, see for example References 45 and 74, methods that provide exactly divergence‐free velocities allow to derive an error estimate that reads as

$$\begin{array}{ll} \|u-u_{h}{\|}_{H^{1},h}\leq c\underset{v_{h}\in V_{h}^{k_{u}}}{\inf}\|u-v_{h}{\|}_{H^{1},h}+F(u), & \; \end{array}$$

where *F*(*u*) is a function that only depends on the exact solution *u* (and not ν) and shows optimal convergence properties (for methods yielding exactly divergence‐free solutions we have *F*(*u*)=0). Hence, these methods show no bad behavior for small values of the viscosity ν. Note that above estimate assumes an exact geometry representation Γ_*h*_=Γ, compare the discussion below.

**Figure 8:**
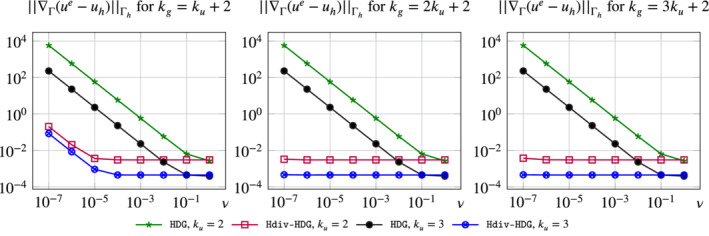
The *H* ^1^‐seminorm error of the velocity solutions of the Stokes problem of section 4.2 for the $H({\text{div}}_{\Gamma })$‐conforming HDG method and the HDG method given by (21) for varying viscosities *ν*=1,…,10^−7^ on a fixed triangulation with geometry approximation order *k*_*g*_=*ik*_*u*_+2 for *i*=1,2,3 [Color figure can be viewed at wileyonlinelibrary.com]

In Figure 8 the *H* ^1^‐seminorm error of the solution of the same problem as above for varying viscosities ν=10^−6^,…,1, two different polynomial orders *k*_*u*_=2,3 and different geometry approximation orders *k*_*g*_=*ik*_*u*_+2 for *i*=1,2,3 is given. We can make several observations. First, the errors of the weakly divergence‐free HDG method, see Equation (21), show the expected behavior: Reducing the viscosity ν leads to a blow up of the *H* ^1^‐seminorm error of the velocity independently of the geometry approximation. We can clearly see the scaling 1/ν for all combination of approximation orders *k*_*u*_ and geometry approximations *k*_*g*_. Next, note that the $H({\text{div}}_{\Gamma })$‐conforming method is expected to be pressure robust as the discrete velocity is exactly divergence‐free. As we can see, this is indeed true if the geometry approximation is accurate enough. In the case *k*_*g*_=3*k*_*u*_+2 the error is constant and independent of the choice of ν. Reducing the approximation order *k*_*g*_ shows that the error is again affected by a change of the viscosity, however the error is still much better compared to the standard HDG method. This behavior is also known from the flat case, and the problem comes from an inexact evaluation of the right‐hand side integral

$$\begin{array}{ll} \int_{\Gamma_{h}}f\cdot v_{h}dx. & \; \end{array}$$

As all integrals are computed using a quadrature rule the right‐hand side integral might not vanish in the case where *f*=∇_Γ_θ and *v*_*h*_ is divergence‐free, that is, ${\text{div}}_{\Gamma }(v_{h})=0$, compare Section 3.5. Beside choosing a high‐order quadrature rule, we now also have to increase the geometry approximation to ensure that a gradient field *f*=∇_Γ_θ is also a gradient field on Γ_*h*_, otherwise again the integral might not vanish even if a high order quadrature rule is used.

### 4.3 Generalization of the Schäfer Turek benchmark for surfaces

We consider the setup of a standard benchmark test case, cf Schäfer et al^75^ [case 2D‐2]. First we recall the setup in the plane and afterwards apply different mappings to obtain geometries in 3D, some of them as in Fries.^38^

#### 4.3.1 The flat setup (reference configuration)

**Figure 9:**
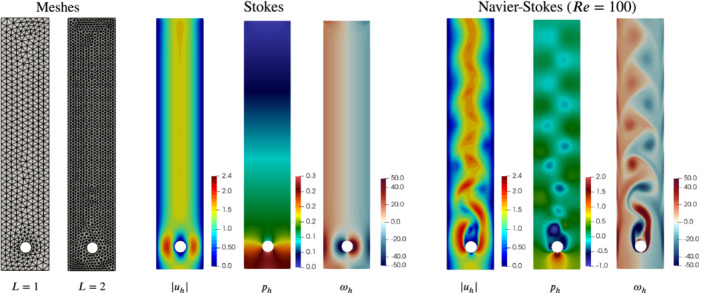
Flat Schäfer‐Turek benchmark problem 2D‐2. The first two meshes used in the simulations (left), discrete solution of the Stokes problem (center), and discrete solution of the unsteady Navier‐Stokes problem at a fixed time (right) [Color figure can be viewed at wileyonlinelibrary.com]

The domain is a rectangular channel without an almost vertically centered circular obstacle, cf Figure 9,

$$\hat{\Gamma }:=[0,2.2]\times [0,0.41]\setminus B_{0.05}((0.2,0.2)).$$

We denote the velocity and pressure solution as û and $\hat{p}$. The boundary is decomposed into ${\hat{\gamma }}_{\text{in}}:=\{\hat{x}=0\}$, the inflow boundary, ${\hat{\gamma }}_{\text{out}}:=\{\hat{x}=2.2\}$, the outflow boundary and ${\hat{\gamma }}_{W}:=\partial \Gamma \setminus ({\hat{\gamma }}_{\text{in}}\cup {\hat{\gamma }}_{\text{out}})$. On ${\hat{\gamma }}_{\text{out}}$ we prescribe natural boundary conditions which read as $(-2\nu \varepsilon_{\Gamma }(û)+\hat{p}P)\cdot \hat{\mu }=0$; on ${\hat{\gamma }}_{W}$ homogeneous Dirichlet boundary conditions for the velocity and on ${\hat{\gamma }}_{\text{in}}$ the inflow Dirichlet boundary conditions

$$û(0,y,t)={û}_{D}=ū\cdot 6\cdot y(0.41-y)/0.41^{2}\cdot (1,0,0).$$

Here, ū=1 is the average inflow velocity and the viscosity is fixed to ν=10^−3^ which results in a Reynolds number *Re*=100. This setup results in a time‐periodic flow with vortex shedding behind the obstacle, cf Figure 9.

The (time dependent) quantities of interest in this example are the forces that act on the disc ${\hat{\gamma }}_{\circ }=S_{0.05}((0.2,0.2))$ and the pressure difference before and behind the obstacle:

$$F_{\circ }:=\int_{{\hat{\gamma }}_{\circ }}\sigma (u,p)\cdot \hat{\mu }dŝ,\;\Delta p:=p_{\text{front}}-p_{\text{back}},p_{\text{front}}=p(0.15,0.5),\;p_{\text{back}}=p(0.25,0.5).$$

This benchmark problem is well studied in the literature and reference values are available in Schäfer et al.^75^ Below, we consider similar setups on geometries $\Gamma_{i}=\Phi_{i}(\hat{\Gamma })$ for mappings Φ_*i*_,*i*=1,…,4 specified below. For *b*∈{in,out,*W*,∘} we define the boundary segments accordingly, that is, $\gamma_{b}:=\Phi_{i}({\hat{\gamma }}_{b})$ and prescribe natural outflow conditions on γ_out_, homogeneuous Dirichlet conditions on γ_*W*_ and prescribe inflow velocities on γ_in_. For the inflow velocities we take (for sake of comparability) the choice of Fries^38^: $u{|}_{\gamma_{\text{in}}}=\Phi_{i}^{\prime }\;û\circ \Phi_{i}^{-1}$. Let us stress that this results in a tangential velocity, but may result in an average inflow velocity that may deviate from ū=1. We note that the degrees of freedoms of our $H({\text{div}}_{\Gamma })$‐conforming HDG formulation naturally fit the tangential boundary conditions. The inflow which is in conormal direction **μ** corresponds to the degrees of freedom of the $V_{h}^{k}$ whereas tangential flow conditions (here: zero) correspond to the boundary degrees of freedom of $\Lambda_{h}^{k_{u}}$.

#### 4.3.2 Computational setup

We solve the surface Navier‐Stokes problem for four different mappings, but use—for the most part—the same computational setup that we want to describe here first. As initial conditions for the Navier‐Stokes problem, we take the solution of a Stokes problem with the same boundary data. To make sure that the simulation reached the time where the periodicity is established we simulate the problem for 30 time units. For the time stepping we use a second‐order implicit‐explicit (IMEX) time stepping method and consider up to three different time step sizes: $\Delta t=2^{1-L_{t}}\cdot 10^{-3},L_{t}\in \{1,2,3\}$. We consider up to three mesh levels with a characteristic mesh size (w.r.t. the reference configuration) $ĥ=2^{1-L_{s}}\cdot 0.05,L_{s}\in \{1,2,3\}$ corresponding to mesh levels *L*_*s*_=1,2,3. For the nonisometric mapping in Section 4.3.2 we use the same unstructured meshes as in the flat case, resulting in 857 (*L*_*s*_=1), 3179 (*L*_*s*_=2), and 13 081 (*L*_*s*_=3) triangular elements, cf Figure 9 for the first two levels. The meshes are not specifically adapted to improve the approximation of potential boundary layers. For the isometric mappings in Section 4.3.1 we consider only one mesh with 865 elements. In all examples in this section we fix *k*_*u*_=4 and *k*_*g*_=5.

#### 4.3.3 Isometric mappings

**Figure 10:**
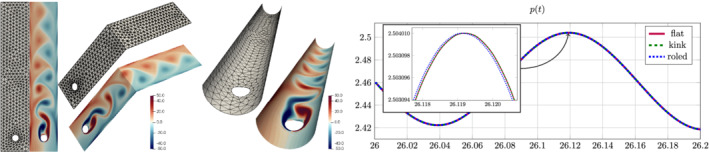
Meshes and vorticity for id (flat), Φ_1_(kink), and Φ_2_(rolled) and pressure difference over time [Color figure can be viewed at wileyonlinelibrary.com]

First, we consider two mappings Φ_*i*_,*i*=1,2 that are isometric, that is, there holds $\hat{d}(\hat{x},ŷ)=d_{\Gamma_{i}}(\Phi_{i}(\hat{x}),\Phi_{i}(ŷ)),\hat{x},ŷ\in \hat{\Gamma }$ where $\hat{d}$ is the two‐dimensional Euclidean distance and *d*_Γ_ is the geodesic distance on the surface $\Gamma_{i}=\Phi_{i}(\hat{\Gamma })$ or equivalently $\det(F_{i}^{T}\cdot F_{i})=1$ with $F_{i}=\Phi_{i}^{\prime }$. Similar to the setup in Section 4.1.3 we consider as a first case

$$\Gamma_{1}=\Phi_{1}(\hat{\Gamma })\;\text{with}\;\Phi_{1}(\hat{x},ŷ)=\left\{\begin{array}{ll} \left(\hat{x}\cdot W,ŷ,H\cdot \hat{x}\right) & \text{if}\;\hat{x}\leq 1.1,\\ \left(\hat{x}\cdot W,ŷ,H\cdot (2.2-\hat{x})\right) & \text{if}\;\hat{x}>1.1, \end{array}\right.$$

with $H=\frac{1}{2}$, $W=\sqrt{3/4}$. To consider the kink in the geometry properly we use a slightly different mesh in this setup than in all others of this subsection by making sure that the line $\hat{x}=1.1$ corresponds to a mesh line, cf Figure 10. Thereby the mapping Φ_*i*_ can be represented exactly in a finite element space so that the mapped mesh introduces no additional geometrical error. We note that already the mesh for $\hat{\Gamma }$ includes (small) approximation errors due to the approximation of the circle.

As a second example we consider

$$\Phi_{2}(\hat{x},ŷ)=(-\frac{0.41}{\pi }\cos(\pi \frac{ŷ}{0.41}),\frac{0.41}{\pi }(1-\sin(\pi \frac{ŷ}{0.41}))),$$

which corresponds to a bending of the flat geometry around the *x*‐axis. This time, when projecting into a finite element space, we can not represent Φ_2_ exactly, hence the isometry property will only be fulfilled approximately. In Figure 10 we show the geometries, the vorticity at a fixes time (*t*=26) and plots that compare Δ*p*(*t*) in a small range of time (*t*∈[26,26.2] on the coarsest mesh level *L*_*s*_=1 with time level *L*_*t*_=2.

We observe that there is hardly any difference between the results. The differences between the results for $\hat{\Gamma }$ and Γ_1_ are only due to round‐off errors and not visible even after heavily zooming in. For Γ_2_ we also only observe differences in the sixth digit.

#### 4.3.4 Nonisometric mapping

As another example we consider a nonisometric mapping, that is, a flow that (not only discretely) deviates from the flat case. The example is one of two similar examples in Fries.^38^ We note that we also tried the other case, but do not present it here due to the similarity in the results. The mapping is

$$\begin{array}{ll} \Phi_{3}(\hat{x},ŷ) & =\left(\cos\left(\pi \frac{\hat{x}}{2.2}\right)\cdot f(ŷ),\;\sin\left(\pi \frac{\hat{x}}{2.2}\right)\cdot f(ŷ),\;2+0.5f(ŷ)-\sin(3f(ŷ))\right),\;\text{with}\;f(ŷ)=ŷ+0.35.\; \end{array}$$

**Figure 11:**
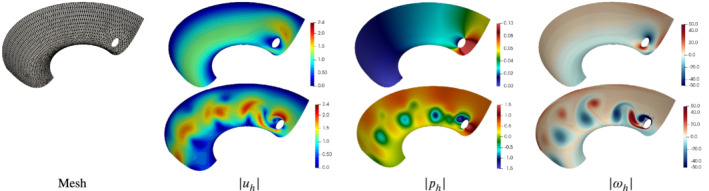
Geometry and surface Stokes (top row) and Navier‐Stokes solution (bottom row) for mapping Φ_3_ [Color figure can be viewed at wileyonlinelibrary.com]

**Figure 12:**
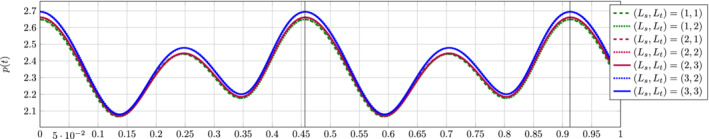
Evolution of pressure difference for mapping Φ_3_ for two periods (starting with a pressure maximum) for different spatial can temporal resolutions [Color figure can be viewed at wileyonlinelibrary.com]

In Figure 11 we display the solution of the surface Stokes and the surface Navier‐Stokes problem. Already for the Stokes problem we see a significant deviation from the flat Stokes solution, especially in the pressure. This is partially due to a different inflow profile, but mainly due to a different length, width and shape of the channel. The vortex shedding behind the obstacle shows a qualitatively similar behavior to the flat case. However, the frequency and the forces acting on the obstacle are different. In the simulation, after 10 time units the vortex shedding is close to periodic. In Figure 12 we display the pressure difference for two periods ($t^{*}=0$ corresponds to a maximum of the pressure within each simulation) for different mesh and time levels. On the finest resolution we obtain a minimum and maximum pressure difference of 2.07982 and 2.69418 which is in very good agreement with the findings in Fries.^38^ Also the period length, which is approximately 0.456343 second (corresponds to a frequency of 2.191334) is in very good agreement with the reference.

### 4.4 The Kelvin‐Helmholtz instability problem on surfaces

In this section we consider the famous Kelvin‐Helmholtz instability problem, cf Reference 48 and the references therein, which is typically defined on the unit square (periodic in *x*‐direction), that is, in 2D. We generalize it to 2D surfaces in 3D. To this end we solve the unsteady Navier‐Stokes equation. In contrast to the previous examples we do not rely on reference domains and mappings, but start directly from surface meshes that are obtained from the mesh generator.

#### 4.4.1 The general setup

In the following examples we consider geometries that are rotational invariant around the *z*‐axis and use a coordinate system (ξ,η) on the surfaces with $\xi \in [-\frac{1}{2},\frac{1}{2})$ following the rotational direction and $\eta \in [-\frac{1}{2},\frac{1}{2})$ perpendicular. Note that ξ and η are not normalized, that is, ‖∇_Γ_ξ‖≠1, ‖∇_Γ_η‖≠1. *e*_ξ_=∇_Γ_ξ/‖∇_Γ_ξ‖, and *e*_η_=∇_Γ_η/‖∇_Γ_η‖ denote the corresponding unit vectors (in the tangential plane of Γ_*h*_) and *r*=*r*(η) denotes the distance (in the ambient space) to the *z*‐axis. The Kelvin‐Helmholtz instability is driven solely by its initial condition. These are taken as

(22) $$\begin{array}{ll} u_{0}(\xi ,\eta ) & =H_{s}(\eta )\cdot u_{\infty }\cdot \frac{r}{R}\cdot e_{\xi }+c_{n}{\mathrm{curl}}_{\Gamma }\psi ,\;\\ \;\text{with}\;H_{s}(\eta ) & :=\tanh\left(\frac{2\eta }{\delta_{0}}\right),\psi (\xi ,\eta ):=u_{\infty }\exp\left(-\frac{\eta^{2}}{\delta_{0}^{2}}\right)\left(a_{a}\cos(m_{a}\pi \xi )+a_{b}\cos(m_{b}\pi \xi )\right), \end{array}$$

the constants *a*_*a*_,*m*_*a*_,*a*_*b*_,*m*_*b*_,*c*_*n*_,δ_0_≥0, and *R* the radius at *z*=0. This means that for *z*>δ_0_ ($H_{s}\approx 1,{\mathrm{curl}}_{\Gamma }\psi \approx 0$) the velocity field corresponds to a rigid body rotation around the *z*‐axis in positive ξ‐direction whereas for *z*<−δ_0_ the velocity corresponds to a rigid body rotation in the opposite direction. The tanh term realizes a smooth transition of the velocity in the intermediate layer which is determined by δ_0_. The terms related to ${\mathrm{curl}}_{\Gamma }\psi$ are a perturbation with the purpose to trigger an instability in a deterministic way. The setup of the problem leads to a number of vortices forming along the shear layer. These vortices eventually pair up to form fewer but larger vortices. In this setup we consider the following three usually investigated global quantities of interest (gradients and curls are to understood in a broken sense, that is, elementwise):

$$\begin{array}{llllllll} \text{Enstrophy:} & \; & ℰ(t) & :=\frac{1}{2}\|\omega_{h}(t){\|}_{L^{2}(\Gamma_{h})}^{2}\; & \text{with vorticity}\;\omega_{h}(t) & :={\mathrm{curl}}_{\Gamma }u_{h}(t), & \; & \;\\ \text{Kinetic energy:} & \; & 𝒦(t) & :=\frac{1}{2}\|u_{h}(t){\|}_{L^{2}(\Gamma_{h})}^{2}, & \text{Palinstrophy:}\;𝒫(t) & :=\frac{1}{2}\|\nabla_{\Gamma }\omega_{h}(t){\|}_{L^{2}(\Gamma_{h})}^{2}.\; & \; & \; \end{array}$$

#### 4.4.2 Computational setup

For the simulations discussed below we use—unless stated otherwise—the same configuration. We fix the intermediate layer size δ_0_=1/28 fix ν=δ_0_/*Re* with *Re*=1000 and set the perturbation constants to *a*_*a*_=1,*m*_*a*_=8,*a*_*b*_=1,*m*_*b*_=20, *c*_*n*_=10^−3^. With the reference time $t_{\text{ref}}:=\frac{u_{\infty }}{\delta_{0}}$ we introduce the scaled time $\bar{t}=t/t_{\text{ref}}$. For the computations we consider a triangular unstructured mesh with characteristic mesh size *h*=0.05, *k*_*u*_=8, *k*_*g*_=9, use the second‐order IMEX time stepping method as before with Δ*t*=10^−3^ *t*_ref_ and simulate until *T*=200*t*_ref_. For α in the SIP stabilization we take 10 again. To initialize the velocity we use a Helmholtz projection in order to make sure that the discrete initial velocity is already exactly divergence‐free, that is, in (16c) we consider *u*_*h*_,*v*_*h*_ in the divergence‐free subspace of $V_{h}^{k_{u}}$, that is, $\left\{v_{h}\in V_{h}^{k_{u}}:b(v_{h},q_{h})=0\;\forall q_{h}\in Q_{h}^{k_{u}-1}\right\}$.

#### 4.4.3 Piecewise smooth manifolds

In this subsection we consider four similar cylindrical setups given by:

$$\begin{array}{ll} \Gamma_{0} & :=\{x\in R^{3}|\|(x,y)\|=R,|z|\leq 1/2\},\\ \Gamma_{1} & :=\Gamma_{0}\cup \{x\in R^{3}|\|(x,y)\|\leq R,|z|=1/2\},\\ \Gamma_{2} & :=\{x\in R^{3}|\|(x,y)\|=R,|z|\leq 1/2-R\}\cup \{x\in R^{3}|\|(x,y)\|\leq R,|z|=1/2-R\},\\ \Gamma_{3} & :=\{x\in R^{3}|\|(x,y)\|=R,|z|\leq 1/4\}\cup \{x\in R^{3}|\|(x,y)\|\leq R,|z|=1/4\}.\; \end{array}$$

The first setting, Γ_0_ is an open cylinder of height 1 with radius *R*=(2π)^−1^, that is, perimeter 1 and we can isometrically map the unit square (periodic in *x*‐direction) on Γ_0_. On the boundary we prescribe free slip boundary condition. As the surface Navier‐Stokes equations are invariant under isometric maps we know that the solution to the corresponding 2D Kelvin‐Helmholtz problem is identical. We can hence compare our numerical solution on Γ_0_ to the results in the literature.^48^

**Figure 13:**
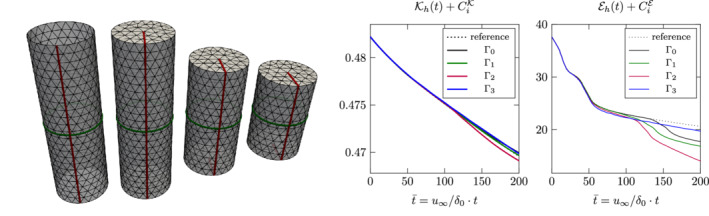
Geometries Γ_*i*_,*i*=0,1,2,3 and corresponding meshes (left). Red lines correspond to *ξ*=0, green lines correspond to *η*=0. Plots of decay of kinetic energy and enstrophy (right). For comparability offsets $C_{i}^{𝒦}$, $C_{i}^{ℰ}$, *i*=0,1,2,3 are added to compensate for differences in initial kinetic energy and initial enstrophy. With $C_{0}^{𝒦}=0$, $C_{1}^{𝒦}=-\frac{1}{8\pi }$, $C_{2}^{𝒦}=\frac{3}{8\pi }$, $C_{3}^{𝒦}=-\frac{1}{8\pi }+\frac{1}{4}$, and $C_{0}^{ℰ}=0$, $C_{1}^{ℰ}=C_{2}^{ℰ}=C_{3}^{ℰ}=-4\pi$ the plotted quantities are equal (up to resolution differences) at time *t*=0 [Color figure can be viewed at wileyonlinelibrary.com]

**Figure 14:**
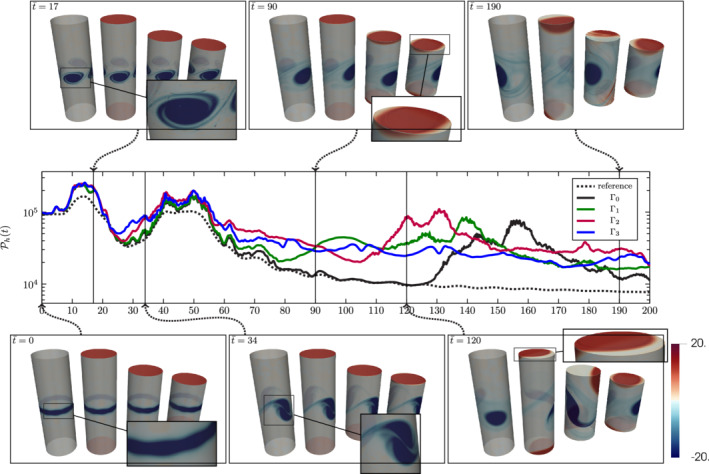
Evolution of ${𝒫}_{h}$ for Γ_*i*_,*i*=0,1,2,3 and the 2D reference solution with vorticity snapshots [Color figure can be viewed at wileyonlinelibrary.com]

The second configuration, Γ_1_, is a closed cylinder with bottom and top added, that is, without boundary. Γ_2_ is similar to Γ_1_ except for the decreased height of 1−2*R*. Hence, the geodesics from the center of the top of the cylinder to the center of the bottom of the cylinder have length 1. The last case, case 3 considers an even shorter closed cylinder with height $\frac{1}{2}$. In Figure 13 the geometries and used meshes are sketched alongside with the decay of energy and enstrophy over time whereas in Figure 14 we plot the palinstrophy alongside a few sketches of the vorticity at selected times.

##### 4.4.3.1 Discussion of results

We observe that the energy dissipation is similar for all cases (up to a constant shift due to different initial kinetic energies). Especially, the kinetic energy is monotonely decreasing. The same holds for the enstrophy for *t*≤100. For later times the results deviate significantly. The reason for those deviations can be best explained by the palinstrophy evolution in Figure 14. Until $\bar{t}=100$ the simulations of the four cases agree very much, at least qualitatively. Initially four vortices form which eventually merge to two vortices around $\bar{t}\in (30,60)$. Until that point of the evolution the rigid body rotations in the most upper and most lower part are essentially not influenced by the interactions in the center. This changes at around $\bar{t}=90$ where the rotations of the top and bottom are perturbed for Γ_2_ and Γ_3_ and somewhat later (around $\bar{t}=120$) also for Γ_1_. For Γ_1_ and Γ_2_ this results in the merging of the latest two vortices to one vortex which is also reflected by an increase in the palinstrophy. For Γ_3_ the perturbation of the rotations seems to stay confined and the decreased height seems to suppress the interaction of the vortices so that even at $\bar{t}=200$ the latest two vortices did not merge yet.

Let us note that we also observe a deviation of the evolution computed for Γ_0_ and the reference solution, especially after $\bar{t}=130$ which is in agreement with the extreme sensitivity of the problem to (numerical) perturbations observed in Reference 48. The final merge is typically observed sooner the higher the perturbations in the simulation are. In contrast to the 2D reference solution we consider a much coarser, not structured and not symmetric mesh with an additional geometry error due to the curved representation.

#### 4.4.4 Smooth manifold: the sphere

Now, we consider the Kelvin‐Helmholtz problem on a smooth manifold, the unit sphere, Γ_4_=*S*_1_(0). The upper and lower halfs rotate in different directions with a perturbation similar as before in (22). However, we change the perturbation magnitude to *c*_*n*_=2π·10^−3^ (due to the increase of the equador length from 1 to 2π) and choose a different initial perturbation mode with *m*_*a*_=16, *a*_*a*_=1, *m*_*b*_=20, and *a*_*b*_=0.1.

**Figure 15:**
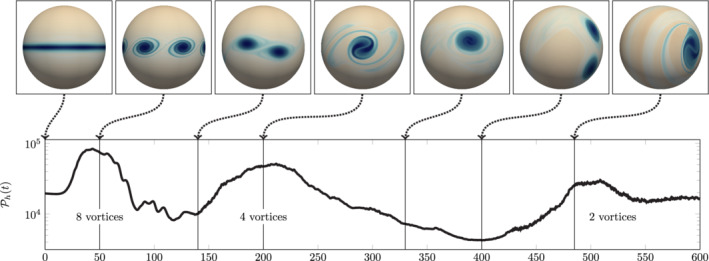
Evolution of ${𝒫}_{h}$ for the Kelvin‐Helmholtz problem on the sphere $\bar{t}\in \{0,50,140,200,330,400,485\}$ [Color figure can be viewed at wileyonlinelibrary.com]

We use *k*_*u*_=5, *k*_*g*_=6 on a unstructured mesh consisting of 5442 triangles. A few snapshots of the flow and the evolution of the palinstrophy are depicted in Figure 15. We observe that initially eight vortices form from the initial perturbation at around $\bar{t}=50$. Diffusion takes its time until it drives the interaction of two neighboring vortices which pair up to four vortices at around $\bar{t}=200$. These vortices take even longer to eventually pair up to two vortices at around $\bar{t}=500$. The two vortices are positioned on opposite sides rotating in opposite directions. The overall evolution of the palinstrophy and the times where vortices pair up is very similar to those from the cylindrical setup in the previous section. However, due to the larger length scales the interaction between the vortices takes more time.

### 4.5 Stanford bunny

**Figure 16:**
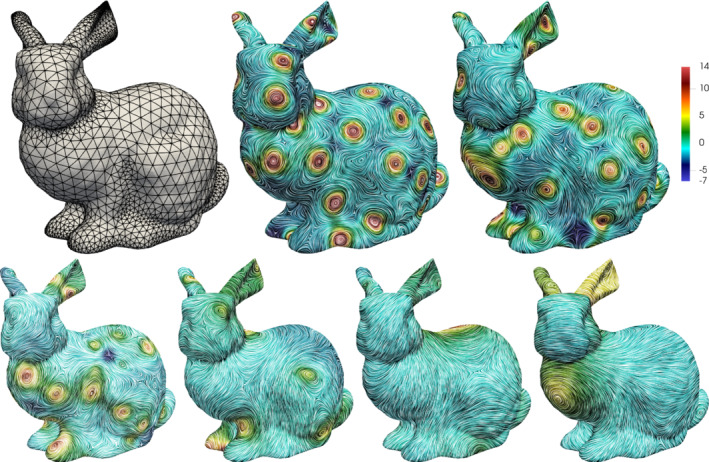
Geometry and mesh of the Stanford bunny (upper left picture) and velocity streamlines with pressure coloring for *t*∈{4,32,60,120,250,500} [Color figure can be viewed at wileyonlinelibrary.com]

As a final example we want to demonstrate that the aforementioned methods can be applied on essentially arbitrary geometries. As a prototype of a complex geometry we take the famous Stanford bunny which fits approximately in a bounding box of size 80×80×60 and initially put 60 vortices on the surface. Let us note that in Reuther and Voigt^32^ this geometry has also been considered (with different initial data) with the aim to investigate defect configurations which is not the aim here. The initial velocity is taken as the surface curl of

$$\psi (x)=20\cdot \overset{60}{\underset{i=1}{\sum }}{\left(-1\right)}^{i}\exp(-\frac{1}{20}\|x-x_{i}{\|}^{2})$$

where the *x*_*i*_,*i*=1,..,60 are some randomly located but sufficiently separated (‖*x*_*i*_−*x*_*j*_‖_2_≥3.5,*i*≠*j*) vertices of the mesh. For the viscosity we choose $\nu =\frac{1}{50}$. The flow is again only driven by its initial condition. Pictures of the numerical solution on a mesh with 6054 triangles, *k*_*u*_=*k*_*g*_=4, time step size $\Delta t=\frac{1}{200}$ and α=80 are shown in Figure 16.

We observe that the flow undergoes a process of self‐organization as it is well‐known from 2D flows. At *t*=*T*=500 the vortices merged successively into two remaining vortices: One around the ears of the Standford bunny, one on the breast. In contrast to the studies in Reuther and Voigt,^32^ the flow is not yet in a quasistationary state at *t*=500, that is, these two vortices will continue moving and possibly merge while the magnitude of the velocity will decay over time.

## 5 CONCLUSION AND OUTLOOK

In this work we introduced new numerical methods for the discretization of incompressible flows and vector‐valued elliptic problems on two‐dimensional manifolds. Abandoning the *H* ^1^‐conformity (originally demanded by the considered problems) we applied the Piola transformation to construct finite elements which are exactly tangential. Based on these findings we presented non‐(*H* ^1^(Γ)‐)conforming (hybrid) DG discretizations which performed extremely well in several considered numerical examples. Among other benefits, it was shown that the resulting methods can outperform *H* ^1^‐conforming discretizations in the aspect of computational costs, and that they can deal with piecewise smooth manifolds which is a unique property so far.

For incompressible flow problems the (hybrid) DG methods were modified to be $H({\text{div}}_{\Gamma })$‐conforming. The resulting methods provide important properties such as exactly (surface) divergence‐free velocity fields, energy stability, and pressure robustness. We studied certain numerical examples showing that the methods are highly accurate and applicable to deal with complex geometries. In particular we also showed a robustness of the new methods with respect to isometric mappings.

The extension of the discretizations to nonstationary (evolving) manifolds is left for future research. Further, due to the increasing interest on foldable LC displays and flows on cell membranes, the coupling of the presented discretizations with other PDEs on surfaces is an interesting task for the future.

## Supporting information

Data S1: Supplementary Information
