# Supplementary material for: Divergence‐free tangential finite element methods for incompressible flows on surfaces
Source: Int J Numer Methods Eng. 2020 Feb 18;121(11):2503–33. doi: 10.1002/nme.6317 (PMC8611805; doi:10.1002/nme.6317)
Supplement: Supplementary file 1 — Data S1: Supplementary Information [file NME-121-2503-s001.docx]

**Small area estimation of receiver operating characteristic curves**

**for ordinal data under stochastic ordering**

**(Manuscript ID: SIM-18-0570)**

This paper proposed a hierarchical Bayesian model to estimate the proper ROC curve and AUC, using stochastic ordering in several domains when the outcome of the diagnostic test is discrete ordinal data. The basic idea and its application are interesting, and the manuscript is well written, but some confusion leaves me concerned about justification for the proposed model.

Comments

1. The stochastic ordering looks like an important feature of the proposed model as seen in the title. However, the authors didn’t explain this in detail. For example, when two populations have stochastic ordering relation, how are two distribution functions defined? The authors need to introduce construction of stochastic ordering proposed by Gelfand and Kuo (1991), and Gelfand and Kottas (2001).

⇒ We have added the explanation for the stochastic ordering as:

*The stochastic order of two populations is defined as:* $F_{1}$ *is stochastically larger than* $F_{0}$ *if* $F_{0}(t)\geq F_{1}(t)$ *for all* $t$ *(Gelfand and Kuo, 1991; Gelfand and Kottas, 2001).*

2. The proposed model incorporated the stochastic ordering because the empirical ROC curve can have a non-concave shape or a hook even when the true ROC curve of the population is a proper ROC curve. So, it is better to compare the model without the stochastic ordering to the proposed model with the stochastic ordering in real data and simulation studies. Doing this can provide more justification of using the proposed model.

⇒ We compared the model without the stochastic ordering to the proposed model with the stochastic ordering in real data analysis and simulation studies. The model without the stochastic ordering can estimate the empirical ROC curve with a non-concave shape or a hook when the true ROC curve of the population is a proper ROC curve. Therefore the model with stochastic ordering is preferred over the model without stochastic ordering.

3. On page 3, in equation (2), there is no explanation or definition about $\rho_{i}$ .

⇒ We have added the explanation or definition about $\rho_{i}$ as:

*We take* $\mu_{ij}\sim N\left( \theta_{i},\delta_{i}^{2} \right)$ *and assume* $\frac{\delta_{i}^{2}}{\sigma^{2}+\delta_{i}^{2}}=\rho_{i}$ *(*$0<\rho_{i}<1)$ *because using* $\rho_{i}$ *based* $(0,1)$ *instead of* $\delta_{i}^{2}$ *with* $0<\delta_{i}^{2}<\infty$ *is helpful for computation.*

4. On page 3, line 38, for the Cauchy prior for $\theta_{i}$ , “$\pi$” in denominator is confused with $\pi$ in $\pi(\theta_{i})$. Please use other letter for probability or vice versa.

⇒ We have changed the notation for the probability distribution to “$p$” instead of “$\pi$”.

5. On page 3, lines 38-45, it needs to clarify why such priors are used in this setting. For instance, why was the standard logistic distribution used instead of standard normal distribution?

⇒ We have added the reasons for the selection of the priors as follows:

*The hyperpriors for* $\theta_{1}$ *and* $\theta_{2}$ *are assumed to be Cauchy prior to consider heavy tailed prior distribution,*

*the hyperprior for* $\sigma^{2}$ *is assumed to be the shrinkage prior to avoid difficulties associated with improper priors and the hyperpriors for* $\rho_{1}$ *and* $\rho_{2}$ *are assumed to be a uniform prior as noninformative prior. We assume the standard logistic distribution with location 0 and scale 1 as a prior for the boundaries because the standard logistic distribution can be transformed into* $U(0,1)$ *in a single way.*

6. On page 4, line 16, it needs to explain how $F_{0}$ and $F_{1}$ are defined to ensure a proper ROC curve. Also, why was the standard normal assumed for $F_{0}$ and $F_{1}$ ? Isn’t it related to discrete ordinal data?

⇒ The ROC curve is proper when $F_{0}(t)\geq F_{1}(t)$.

To satisfy this condition, we define two CDFs as:

$F_{0}\left( \nu_{k} \right)=\Phi\left( \frac{\nu_{k}-\mu_{1j}}{\sigma} \right), F_{1}\left( \nu_{k} \right)=\Phi\left( \frac{\nu_{k}-\mu_{1j}}{\sigma} \right)\Phi\left( \frac{\nu_{k}-\mu_{2j}}{\sigma} \right)$, because $0\leq\Phi(\cdot)\leq1$.

The ROC model with c ordinal response categories assumes that the latent decision-variable axis is partitioned into c categories by c-1 boundaries and we assume the decision-variables in the each domain are distributed normal distribution. In future studies, we will consider another distribution such as the skew –normal distribution.

7. In Section 3.2, why do the small-area model and the individual-area model have different numbers of samples, 160,000 vs. 60,000?

⇒ We have considered different numbers of samples to satisfy the convergence of MCMC algorithm. In the revised manuscript, we have considered the model without the stochastic ordering instead of the individual-area model and used the same numbers of sample.

8. In Section 3.2, it seems better to provide the results from the model without stochastic ordering to improve the proposed model.

⇒ We have compared the model without the stochastic ordering to the proposed model with the stochastic ordering in real data analysis and simulation studies.

9. In simulation study, it is not clear to me how the data are generated. For example, why the actually- positive cases in each domain are always larger than or equal to the actually-negative cases, even though the real data have reversed direction (i.e., the non-appendicitis cases are always larger than the appendicitis cases). Any specific reason?

⇒ We have changed the sample sizes in simulation study. We have considered that the actually-negative and actually-positive cases in each domain are assumed to be (76, 49) because the median number of patients without disease and with disease are shown to be 76 and 49 from a literature survey for studies on diagnostic accuracy (Bachmann et al., 2006).

10. In simulation study, it would be good to make another scenario, in that the model without stochastic ordering provides improper ROC curve, while the proposed model with stochastic ordering provides proper ROC curve. This can enrich the proposed model.

⇒ We have compared the model without the stochastic ordering to the proposed model with the stochastic ordering in simulation studies. We have performed a simulation with a true AUC of 0.6 and a true ROC curve of a proper ROC curve. In the results, some ROC curve in the model without stochastic ordering are located below the 45˚ straight line, which implies the ROC curve is not proper even though the true ROC curve is proper. Therefore, based on simulation results, the model with stochastic ordering is preferred over the model without stochastic ordering.

11. On page 9, is it true that the good acceptance rate is between 25% and 75%? It sounds too wide.

⇒ An acceptance rate of around 50% or slightly lower is ideal. However, a rate somewhere between 25% and 75% is often acceptable (Lynch, 2007). We have changed the sentence as follows:

*To assess whether the MCMC sequence is mixing well, we assess the acceptance rate of the Metropolis algorithm for each parameter and consider that this algorithm works well when the acceptance rate is 50% or slightly lower.*

12. In Figure 1, it is hard to read the plots because the fitted ROC curves and 95% C.Is from two models are made all together. How about keeping ROC curves only?

⇒ We have corrected the graph to have only the fitted ROC curves.

13. A review of the text is needed to correct a few grammar errors.

⇒ We thoroughly reviewed the manuscript and made grammatical improvements.
